# Kidney Transplantation and the Gut–Kidney Axis: Microbial, Metabolic, and Nutritional Implications for Graft and Patient Outcomes

**DOI:** 10.3390/nu18132056

**Published:** 2026-06-24

**Authors:** Leon Smółka, Miłosz Strugała, Karolina Kursa, Karolina Blady, Agata Stanek

**Affiliations:** 1 Student Scientific Association, Department of Internal, Metabolic Diseases and Angiology, Faculty of Health Sciences, Medical University of Silesia, Ziolowa 45/47 St., 40-635 Katowice, Poland; s83152@365.sum.edu.pl (M.S.); s91506@365.sum.edu.pl (K.K.); s86570@365.sum.edu.pl (K.B.); 2 Department of Internal, Metabolic Diseases and Angiology, Faculty of Health Sciences, Medical University of Silesia, Ziolowa 45/47 St., 40-635 Katowice, Poland; 3 Upper Silesian Medical Center, Medical University of Silesia, Ziolowa 45/47 St., 40-635 Katowice, Poland

**Keywords:** kidney transplantation, kidney transplant recipients, gut–kidney axis, gut microbiota, graft function, graft outcomes, post-transplant complications, nutritional modulation, microbiota-derived metabolites, trimethylamine *N*-oxide

## Abstract

Background: Kidney transplantation is the preferred treatment for end-stage kidney disease (ESKD), but long-term outcomes remain limited by chronic allograft injury, infections, metabolic complications, and cardiovascular risk. Gut microbiota alterations and microbiota-derived metabolites may influence immune regulation, inflammation, drug metabolism, and graft outcomes through the gut–kidney axis. This review summarizes evidence on the gut microbiota in kidney transplantation, emphasizing immune tolerance, complications, cardiovascular risk, graft function, and perspectives. Methods: A structured search was conducted in PubMed, Scopus, and Web of Science to May 2026. Eligible publications included studies involving kidney transplant recipients (KTR), kidney disease or solid organ transplant populations, and mechanistic models. Evidence was synthesized narratively. Results: Gut microbiota alterations in KTR reflect pre-transplant dysbiosis and post-transplant exposures, including antibiotics, immunosuppression, infection, diet, hospitalization, and graft function. Dietary factors and nutrient-derived substrates may modulate microbial composition and production of relevant metabolites, including short-chain fatty acids (SCFAs), trimethylamine *N*-oxide (TMAO), tryptophan-derived compounds, bile acid derivatives, and uremic toxins. Microbiota-related pathways may involve barrier dysfunction, microbial translocation, innate immune activation, altered regulatory T cell/T helper 17 (Treg/Th17) balance, metabolite signaling, uremic toxin generation, and endothelial stress. Clinical studies associate dysbiosis and microbial metabolites with diarrhea, infections, delayed graft function (DGF), rejection-related shifts, tacrolimus variability, cardiovascular risk, graft dysfunction, graft failure, and mortality. Most findings need validation. Conclusions: Gut microbiota signatures and microbial metabolites are promising markers of transplant-related risk, but not established causal determinants or therapeutic targets. Clinical translation requires standardized methods, multi-omics integration, and prospective patient- and graft-centered trials.

## 1. Introduction

Kidney transplantation remains the preferred treatment for patients with end-stage kidney disease (ESKD), providing substantial improvements in survival, cardiovascular outcomes, and quality of life compared with maintenance dialysis. Advances in surgical techniques, immunosuppressive strategies, and perioperative management have significantly improved short-term graft survival and reduced the incidence of acute rejection episodes. Nevertheless, long-term graft survival remains limited by chronic alloimmune injury, infections, metabolic disturbances, and persistent cardiovascular risk, which continues to represent the leading cause of mortality after kidney transplantation. These observations highlight the importance of identifying additional biological mechanisms that may influence long-term transplant outcomes beyond classical immunological pathways [1,2].

Increasing evidence suggests that the gut microbiota plays an important role in regulating systemic immune responses, metabolic homeostasis, and inflammatory signaling through mechanisms collectively described as the gut–kidney axis. In patients with chronic kidney disease (CKD), alterations in microbial composition and function develop early and may contribute to uremic toxin accumulation, intestinal barrier dysfunction, and chronic low-grade inflammation. Importantly, these microbiota alterations may persist after kidney transplantation and can be further modified by immunosuppressive therapy, antibiotic exposure, hospitalization, and dietary changes, potentially influencing immune regulation and graft stability in transplant recipients [3,4].

Kidney transplant recipients (KTRs) represent a particularly complex population in whom microbiota-related mechanisms are shaped by both pre-transplant exposures and post-transplant therapeutic interventions. Long-term dialysis therapy, uremia-associated metabolic disturbances, and chronic inflammation contribute to baseline dysbiosis prior to transplantation, whereas calcineurin inhibitors, corticosteroids, antimicrobial prophylaxis, and changes in nutritional status may further influence microbial composition after transplantation. Emerging studies suggest that microbiota-derived metabolites, including SCFAs, trimethylamine *N*-oxide (TMAO), and tryptophan-derived compounds, may participate in pathways related to immune regulation, endothelial function, and inflammatory signaling that are relevant to graft function and vascular risk after transplantation [5,6].

Beyond immune regulation, the gut microbiota has also been associated with several clinically relevant post-transplant complications, including infections, gastrointestinal disturbances, DGF, and variability in immunosuppressive drug metabolism. Increasing attention has also been directed toward microbiota-related pathways that may contribute to persistent cardiovascular risk after transplantation despite restoration of renal function. Recent studies suggest that microbiome-related mechanisms may represent potential targets for biomarker development and future microbiota-modulating interventions aimed at improving long-term transplant outcomes [7,8,9,10]. Therefore, the aim of this review is to summarize current evidence regarding gut microbiota alterations in KTR and to discuss their potential implications for immune tolerance, post-transplant complications, cardiovascular risk, and graft outcomes, with particular attention to microbiota-derived metabolites, nutritional modulation, and emerging microbiome-based diagnostic and therapeutic strategies.

## 2. Materials and Methods

A structured literature search was conducted in PubMed, Scopus, and Web of Science from database inception to May 2026 to identify publications addressing associations between the gut microbiota, microbiota-derived metabolites, nutritional factors, and kidney transplantation. This review was designed as a structured narrative synthesis of clinical, translational, and mechanistic evidence related to gut microbiota alterations in KTR and their potential implications for immune regulation, post-transplant complications, cardiovascular and vascular risk, graft function, and diagnostic or therapeutic strategies. Because of the narrative scope of the review and the heterogeneity of the available evidence, a formal review protocol was not prepared or registered.

The search strategy combined terms related to kidney transplantation, renal transplant recipients, graft outcomes, immunosuppression, and post-transplant complications with terms related to the gut microbiota, dysbiosis, metagenomics, 16S rRNA sequencing, microbial metabolites, intestinal barrier function, microbial translocation, probiotics, prebiotics, synbiotics, fecal microbiota transplantation, nutrition, and pharmacomicrobiomics. The following core search string was used and adapted for each database: (“kidney transplantation” OR “renal transplantation” OR “kidney transplant recipients” OR “renal transplant recipients”) AND (“gut microbiota” OR “gut microbiome” OR dysbiosis OR metagenomics OR “16S rRNA” OR “microbial metabolites” OR “intestinal barrier” OR “microbial translocation”). Additional targeted searches combined transplantation-related terms with (“short-chain fatty acids” OR SCFA OR butyrate OR propionate OR acetate OR TMAO OR trimethylamine *N*-oxide OR choline OR carnitine OR “red meat” OR tryptophan OR indole OR kynurenine OR “bile acids” OR “uremic toxins” OR “indoxyl sulfate” OR “*p*-cresyl sulfate” OR probiotics OR prebiotics OR synbiotics OR “fecal microbiota transplantation” OR nutrition OR diet OR dietary fiber OR “Mediterranean diet” OR “plant-based diet” OR “post-transplant diabetes” OR “weight gain”). The complete search strategies for PubMed, Scopus, and Web of Science, including database-specific syntax, are provided in Supplementary Method S1.

Additional targeted searches were performed for specific clinical outcomes discussed in this review, including diarrhea, *Clostridioides difficile* infection (CDI), urinary tract infection (UTI), DGF, rejection, chronic allograft dysfunction, cardiovascular disease, endothelial dysfunction, arterial stiffness, peripheral arterial disease (PAD), graft failure, and mortality. Records retrieved from databases were deduplicated before screening. Titles and abstracts were screened independently by two reviewers. Potentially eligible full-text articles were then assessed independently by two reviewers, and disagreements were resolved by discussion and consensus; when necessary, a third reviewer was consulted. Data extraction was performed independently by two reviewers using a predefined extraction framework, and discrepancies were resolved by discussion and consensus.

Records were excluded at the title and abstract stage if they were non-English, unavailable as full text, or not relevant to the review topic. During full-text assessment, reports were excluded if they were not relevant to kidney transplantation, gut microbiota, microbial metabolites, nutrition, or microbiota-targeted interventions, or if they were conference papers, letters to the editor, or publications without sufficient relevance to the aims of this review. Reference lists of selected articles and relevant reviews were also screened; however, this did not yield additional unique eligible records beyond those identified in the database search.

Eligible publications included original human observational or interventional studies involving KTR, as well as selected preclinical studies providing mechanistic insight into host–microbe interactions, immune regulation, intestinal barrier function, microbial metabolites, inflammation, or graft-related pathways. Studies in CKD, dialysis populations, or other solid organ transplant recipients were included when they were directly relevant to pre-transplant dysbiosis, transplant-related exposures, microbiota-targeted interventions, biomarker interpretation, or safety considerations. Studies assessing non-gut microbial compartments, such as salivary or urinary microbiota, were included only when they addressed clinically relevant transplant outcomes and were interpreted as complementary evidence rather than direct evidence of gut microbiota involvement.

To improve transparency in the interpretation of evidence, studies were categorized according to the source and level of evidence. Direct transplant evidence was defined as evidence derived from KTR studies assessing gut or stool microbiota, gut microbiome profiles, microbiota-derived metabolites, or nutrition–microbiota interactions. Complementary transplant evidence included studies in KTR that assessed non-gut microbial compartments, such as salivary or urinary microbiota. Indirect clinical evidence included studies in CKD, dialysis populations, or other solid organ transplant recipients. Mechanistic or preclinical evidence included animal, cellular, experimental, or model-based studies addressing biological pathways relevant to host–microbe interactions, immune regulation, intestinal barrier function, microbial metabolites, or graft-related injury. This classification was used throughout the narrative synthesis to distinguish findings directly supported by KTR data from indirect or mechanistic evidence.

Extracted information included study design, population or model, sample size, timing in relation to transplantation, sample type, microbiome assessment method, metabolomic or functional profiling approach, key microbial taxa or pathways, microbiota-derived metabolites, nutritional exposures, transplant-related outcomes, adjustment for confounders, and main limitations. Particular attention was paid to factors that may confound associations between microbiota-related variables and clinical outcomes, including antibiotic exposure, immunosuppressive regimen, diarrhea, infection, hospitalization, dietary intake, renal function or eGFR, diabetes, proton-pump inhibitor use, and time since transplantation. To further address heterogeneity in transplant recipient characteristics, Supplementary Table S1 summarizes whether selected KTR studies reported the underlying CKD or ESKD etiology, autoimmune or immune-mediated kidney disease, background immunological disorders, immunosuppressive treatment, and antibiotic exposure.

The methodological quality of included original human studies was assessed using design-specific critical appraisal tools. Observational cohort, cross-sectional, and case–control studies were evaluated using the appropriate Joanna Briggs Institute critical appraisal checklists. Interventional studies, where applicable, were assessed using relevant design-specific appraisal criteria. Quality assessment was performed independently by two reviewers, and disagreements were resolved by discussion and consensus. Studies were not excluded solely on the basis of methodological quality; instead, methodological appraisal categories were used to contextualize the strength and reliability of the evidence. The results of the methodological quality assessment are summarized in Supplementary Table S2. Overall, most transplant-specific studies were observational and were limited by small or moderate sample size, heterogeneous sampling time, variable microbiome and metabolomic methods, and incomplete adjustment for antibiotics, immunosuppression, diet, renal function, diarrhea, infection, and time after transplantation.

**Figure 1:**
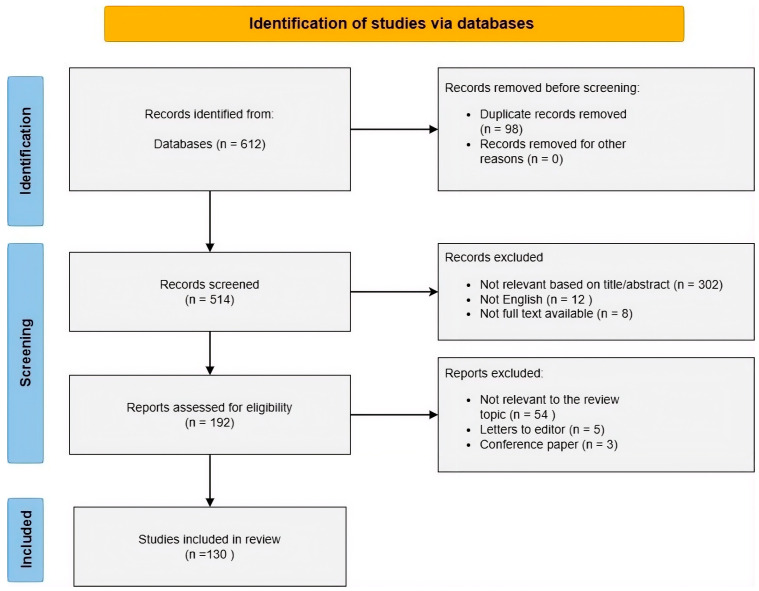
Flow diagram of the literature search and study selection process.

Evidence was synthesized narratively and organized according to the main thematic domains of this review: baseline and post-transplant dysbiosis, immune tolerance and host–microbe interactions, post-transplant complications, cardiovascular and vascular consequences, graft function and outcomes, nutritional modulation, and diagnostic or therapeutic perspectives. Because of substantial heterogeneity in study design, sample type, timing of specimen collection, microbiome and metabolomic assessment methods, analytical platforms, patient populations, immunosuppressive and antibiotic exposures, dietary assessment, and clinical endpoint definitions, quantitative pooling or meta-analysis of effect sizes was not performed. In particular, the available studies differed in whether they assessed KTR directly or provided supportive evidence from CKD, dialysis, other solid organ transplant, salivary or urinary microbiome studies, or mechanistic settings. Therefore, the evidence was synthesized narratively, with emphasis on the strength, source, methodological quality, and limitations of each line of evidence. The study selection process and reasons for exclusion are summarized in the flow diagram presented as Figure 1.

## 3. The Gut Microbiota and Dysbiosis in Kidney Transplant Recipients

KTRs represent a distinct clinical population in whom gut microbiota composition reflects the cumulative influence of CKD-associated metabolic disturbances, dialysis-related factors, together with peri- and post-transplant environmental exposures related to hospitalization, antimicrobial therapy, dietary changes, and immunosuppressive treatment. Increasing evidence indicates that microbiota alterations observed after transplantation frequently originate during earlier stages of renal dysfunction and may persist despite restoration of kidney function after successful graft implantation. Consequently, gut microbiota composition in KTR should be interpreted as a dynamic phenotype shaped by both pre-transplant and post-transplant determinants rather than as a microbial state emerging exclusively after transplantation [5,8].

The evidence summarized in this section includes both direct KTR studies and indirect evidence from CKD or dialysis populations. Direct transplant evidence supports the presence of gut microbiota alterations in KTR, whereas evidence from CKD and dialysis populations mainly helps explain the pre-transplant background from which many recipients enter transplantation. Accordingly, microbiota-related findings in KTR should be interpreted in relation to baseline kidney disease, dialysis exposure, antibiotics, immunosuppression, diet, hospitalization, graft function, and time since transplantation [11,12].

### 3.1. Baseline Gut Microbiota Alterations Before Kidney Transplantation

Most evidence regarding baseline gut microbiota alterations before transplantation comes from CKD and dialysis populations rather than from longitudinal studies following the same individuals from advanced kidney disease through transplantation. CKD is associated with early and progressive alterations in gut microbiota composition that become more pronounced with advancing renal dysfunction. Reduced microbial diversity, enrichment of urease-producing bacterial taxa, and increased abundance of proteolytic microorganisms have been consistently observed in patients with ESKD. These microbiota changes are associated with impaired intestinal barrier integrity, increased intestinal ammonia production, and systemic low-grade inflammation, which together contribute to accumulation of microbiota-derived uremic toxins such as indoxyl sulfate and *p*-cresyl sulfate [13,14].

Dietary restrictions commonly implemented in patients with advanced CKD represent an additional determinant of microbiota alterations prior to transplantation. Reduced intake of dietary fiber and plant-derived nutrients may lower the availability of substrates for SCFA production and reduce the abundance or activity of taxa involved in fiber fermentation. In parallel, repeated antibiotic exposure, delayed intestinal transit, comorbidity burden, and dialysis-related healthcare exposure may further modify microbial composition and metabolic activity [15,16].

Dialysis therapy further contributes to microbiota instability before transplantation through repeated healthcare exposure, metabolic stress, and modality-specific differences related to hemodialysis and peritoneal dialysis. Recent comparative microbiome analyses demonstrate distinct microbial signatures between dialysis patients and KTR, suggesting that baseline dysbiosis acquired during renal replacement therapy may persist into the early post-transplant period [17,18]. However, such comparisons should be interpreted cautiously because dialysis patients and transplant recipients differ in kidney function, medication exposure, diet, comorbidity profile, healthcare contact, and inflammatory status.

### 3.2. Determinants of Post-Transplant Dysbiosis in Kidney Transplant Recipients

Following transplantation, gut microbiota composition undergoes additional modifications driven by perioperative exposures and long-term immunosuppressive therapy. Direct KTR studies indicate that microbiota profiles after transplantation are influenced by timing after transplantation, antimicrobial exposure, immunosuppressive regimen, infection, gastrointestinal symptoms, diet, and graft function. These factors may act simultaneously, making it difficult to attribute specific microbial changes to transplantation itself [19,20].

Calcineurin inhibitors and corticosteroids may influence microbial diversity indirectly through metabolic effects and immune modulation, whereas mycophenolate mofetil has been associated with gastrointestinal symptoms that may further alter intestinal microbial composition. In addition, perioperative antibiotic prophylaxis and repeated antimicrobial treatment during the post-transplant period are associated with reductions in commensal bacterial populations and enrichment of opportunistic taxa [10,19]. Antibiotic exposure should be considered one of the strongest confounders in transplant microbiome studies because it may both reflect clinical complications and directly reshape microbial composition.

Hospitalization-related exposures represent an additional determinant of microbiota instability during the peri-transplant period. Surgical stress, temporary fasting, changes in nutritional patterns, reduced physical activity, and exposure to hospital-associated microbial environments may contribute to transient but clinically relevant microbial shifts. Longitudinal analyses indicate that microbiota diversity may partially recover after transplantation, although persistent dysbiosis has been reported during longer follow-up in a subset of transplant recipients [21,22]. These findings support the need to consider time since transplantation when interpreting microbiota profiles.

**Figure 2:**
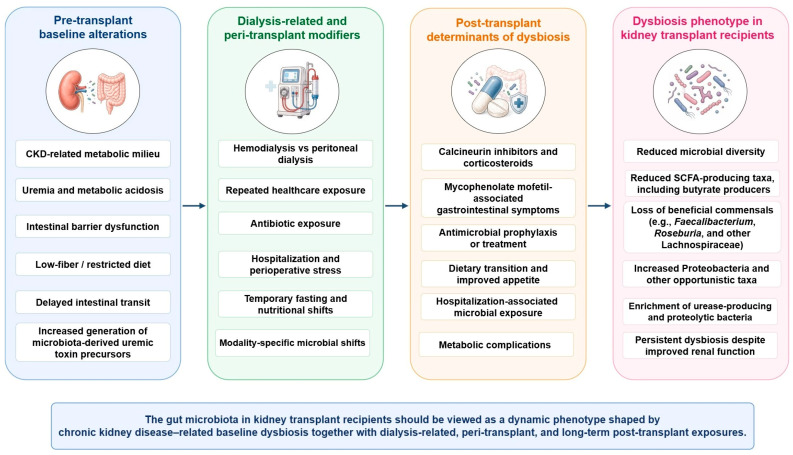
Determinants of gut microbiota dysbiosis before and after kidney transplantation. Gut microbiota composition in KTR reflects cumulative influences acting across the transplant timeline rather than a microbial state emerging exclusively after transplantation. CKD-related metabolic disturbances, dialysis- and peri-transplant modifiers, and post-transplant exposures collectively shape a dysbiosis phenotype characterized by reduced microbial diversity, depletion of SCFA-producing taxa, reduced abundance of taxa such as *Faecalibacterium*, *Roseburia*, and other *Lachnospiraceae*, enrichment of *Proteobacteria* and urease-producing or proteolytic bacteria, and possible persistence of dysbiosis despite improved renal function. Blue boxes indicate pre-transplant baseline alterations, green boxes represent dialysis-related and peri-transplant modifiers, orange boxes denote post-transplant determinants of dysbiosis, and pink boxes show the characteristic dysbiosis phenotype described in KTR. Abbreviations: CKD—chronic kidney disease; SCFA—short-chain fatty acid.

Dietary transitions occurring after transplantation represent another determinant of microbiota composition. Relaxation of pre-transplant dietary restrictions, improved appetite, and changes in macronutrient intake may influence microbial diversity and functional activity during long-term follow-up. In addition, metabolic complications frequently observed after transplantation, including post-transplant diabetes and weight gain, may further contribute to microbiota variability in transplant recipients [3,23]. Taken together, these observations indicate that post-transplant dysbiosis should not be interpreted as the result of a single exposure, but rather as a cumulative and dynamic process shaped by pre-existing CKD-related alterations, dialysis- and peri-transplant factors, and post-transplant therapeutic and environmental influences. The main determinants of gut microbiota dysbiosis across the transplant timeline are summarized in Figure 2.

### 3.3. Taxonomic Features of Gut Dysbiosis in Kidney Transplant Recipients

In addition to reduced microbial diversity, KTRs exhibit characteristic taxonomic shifts reflecting both CKD-related dysbiosis and post-transplant environmental exposures. Several studies have reported reduced abundance of short-chain fatty acid-producing taxa belonging to the genera *Faecalibacterium*, *Roseburia*, and members of the *Lachnospiraceae* family, which are associated with maintenance of intestinal epithelial barrier integrity and metabolic homeostasis. At the same time, enrichment of microorganisms belonging to the phylum Proteobacteria has been observed in transplant recipients, suggesting persistent microbiota instability after transplantation [11,20].

However, these taxa should not be interpreted as universally protective or harmful, because microbial effects are context-dependent and may vary according to host physiology, diet, medication exposure, infection status, and graft function. Therefore, taxonomic shifts should be interpreted as markers of altered microbial ecology rather than as direct evidence of causality.

Expansion of urease-producing bacteria and enrichment of taxa involved in proteolytic fermentation represent additional microbiota features associated with CKD and kidney transplantation. These microbial changes are associated with increased intestinal production of metabolites derived from aromatic amino acids, including precursors of indoxyl sulfate and *p*-cresyl sulfate, and may persist despite improvement in renal function after transplantation [13,14]. However, this evidence is derived largely from CKD and dialysis studies, and its direct clinical relevance after kidney transplantation depends on residual graft function, renal clearance, diet, and host metabolism. To provide a clearer overview of microbial features and their evidence source, selected taxa and microbial groups relevant to kidney transplantation are summarized in Table 1.

## 4. Immune Tolerance and Host–Microbe Interactions

The gut microbiota represents an important interface between the intestinal environment and systemic immune regulation. In KTR, this interface is clinically relevant because alloimmune responses, infection susceptibility, immunosuppressive treatment, intestinal barrier integrity, and microbial metabolism may interact throughout the post-transplant course [33,34]. Therefore, gut microbiota alterations should be regarded as potential modifiers of transplant-related immune responses rather than isolated determinants of graft outcome. The evidence discussed in this section is largely mechanistic or indirect, with limited direct validation in KTR cohorts. Accordingly, the mechanisms described below should be interpreted as biologically plausible pathways that may help explain observed associations, not as proven causal mechanisms of rejection, graft dysfunction, or transplant outcomes. The main mechanisms linking dysbiosis with immune regulation include innate immune signaling, regulatory and effector T-cell balance, intestinal permeability, microbial translocation, and immunomodulatory metabolites [33,35].

### 4.1. Regulation of Immune Responses by the Gut Microbiota: TLR Signaling and the Treg/Th17 Axis

The immunomodulatory role of the gut microbiota has been described across several inflammatory and vascular diseases [36,37]. Microbial signals may influence host immunity through pattern-recognition receptors (PRRs), including Toll-like receptors (TLRs), which recognize conserved microbial-associated molecular patterns [38,39]. Among these ligands, lipopolysaccharide (LPS) can activate the Toll-like receptor 4/myeloid differentiation factor-2 (TLR4/MD-2) receptor complex and downstream inflammatory pathways, including myeloid differentiation primary response 88 (MyD88)-dependent, TIR domain-containing adaptor inducing interferon beta (TRIF)-dependent, nuclear factor kappa-light-chain-enhancer of activated B cells (NF-κB), and activator protein 1 (AP-1)-related signaling [38,39,40,41,42]. In dysbiosis, altered microbial composition, impaired barrier function, and increased exposure to microbial products may shift these pathways toward persistent low-grade immune activation [43,44]. In KTR, this may be relevant because inflammatory signaling can influence antigen-presenting cell maturation, costimulatory pathways, and effector T-cell responses [33,35]. However, most evidence linking gut-derived PRR/TLR signaling directly to graft outcomes in KTR remains extrapolated from mechanistic, inflammatory disease, or non-transplant settings.

A second important immunological interface involves the balance between regulatory T cells and pro-inflammatory T-cell subsets. Forkhead box P3 (Foxp3) regulatory T cells (Tregs) are central to immune tolerance, whereas Th1 and Th17 responses are generally associated with inflammatory and alloimmune activation [45]. Experimental data suggest bidirectional interactions between mucosal immunity and the microbiota, because disruption of Treg function can influence gut microbial composition, while chronic exposure to pro-inflammatory microbial or metabolic signals may promote dendritic cell maturation, antigen presentation, and activation of effector T-cell populations, including Th1 and Th17 cells [46,47,48]. These experimental observations support biological plausibility, but they do not by themselves establish that gut microbiota alterations drive alloimmune injury in KTR.

Clinical transplant data support the relevance of the Treg/Th17 axis in graft rejection. In a flow cytometry-based study, Ma et al. reported that renal transplant recipients with rejection had increased Th17 cells, reduced Treg cells, and altered inflammatory cytokine profiles compared with recipients without rejection [45]. Because this study did not directly assess gut microbiota composition, it supports the Treg/Th17 axis as an immunological interface rather than a microbiome-specific mechanism [45]. Thus, this study provides direct transplant immunology evidence, but not direct gut microbiota evidence.

Beyond graft-centered endpoints, Swarte et al. reported that health-related quality of life in KTR was linked to gut microbiome features, with greater deviation from a healthy microbiome profile associated with worse physical and mental health-related outcomes [49]. Although these findings do not establish an immunological mechanism, they support integration of microbiome research with immunological, metabolic, and patient-reported outcomes in kidney transplant populations. These data should be interpreted as associative clinical microbiome evidence rather than proof of immune-mediated causality.

### 4.2. Intestinal Barrier Dysfunction in Kidney Transplant Recipients

The intestinal barrier maintains selective permeability while limiting translocation of bacteria and microbial products from the gut lumen into the systemic circulation. Structurally, it depends on epithelial cells connected by tight junction proteins, including claudins, occludin, and zonula occludens-1 (ZO-1), which regulate nutrient and solute exchange while restricting excessive microbial translocation [50,51].

In KTR, intestinal barrier function may be affected by both pre- and post-transplant factors. Before transplantation, ESKD and dialysis are associated with uremia, intestinal dysbiosis, and accumulation of gut-derived uremic toxins, which arise through host–microbe metabolic interactions and may be insufficiently cleared in advanced kidney disease [52]. Uremic conditions may further aggravate dysbiosis and impair epithelial barrier integrity through inflammatory, metabolic, and epithelial mechanisms [44]. This pre-transplant evidence is derived mainly from CKD and dialysis populations and should therefore be considered indirect evidence for KTR. After transplantation, partial restoration of kidney function may reduce some uremia-related disturbances, but barrier dysfunction may persist or re-emerge due to residual graft dysfunction, antibiotic exposure, infections, perioperative stress, dietary changes, and immunosuppressive therapy. Impaired barrier integrity may facilitate systemic passage of microbial products, including LPS, contributing to metabolic endotoxemia and chronic low-grade inflammation [43]. In transplant recipients, this inflammatory signaling could theoretically amplify innate immune activation and interfere with immune regulation [33,35]. However, direct longitudinal studies integrating intestinal permeability markers, microbial translocation markers, gut microbiota profiling, and biopsy-confirmed graft outcomes in KTR remain limited.

Overall, intestinal barrier dysfunction represents a plausible mechanistic bridge between dysbiosis and systemic immune activation. Future studies integrating barrier markers, microbial products, immunophenotyping, and biopsy-confirmed graft endpoints are required to clarify its prognostic and therapeutic relevance [33,43,44]. At present, barrier dysfunction should be presented as a biologically plausible pathway rather than a validated mediator of graft injury in clinical transplantation.

### 4.3. Microbial Metabolites and Immune Regulation

Microbial metabolites represent another pathway through which the gut microbiota may influence systemic immunity. In kidney transplantation, SCFAs, tryptophan-derived compounds, bile acid derivatives, and microbiota-derived uremic toxins are of interest because they may affect intestinal barrier function, inflammatory signaling, T-cell differentiation, endothelial dysfunction, and graft-related risk, particularly when renal clearance remains impaired [33,34]. However, circulating and fecal metabolite concentrations should not be interpreted as direct readouts of microbial production alone, because they are also influenced by dietary intake, host metabolism, hepatic processing, graft function, renal clearance, medication exposure, and time after transplantation [53]. This is particularly relevant in KTR, in whom changing graft function may substantially affect metabolite accumulation and clearance. SCFAs, including butyrate, propionate, and acetate, are produced mainly through anaerobic fermentation of dietary fiber by commensal bacteria, including *Faecalibacterium prausnitzii*, *Roseburia* spp., and members of the Lachnospiraceae and Ruminococcaceae families [24]. SCFAs, particularly butyrate, may influence immune responses through inhibition of histone deacetylases (HDACs), reduction in pro-inflammatory cytokine production, support of Treg differentiation or function, and signaling through G protein-coupled receptors, including free fatty acid receptor 3 (GPR41), free fatty acid receptor 2 (GPR43), and hydroxycarboxylic acid receptor 2 (GPR109A) [54,55,56,57]. Butyrate also supports colonocyte metabolism and epithelial barrier integrity [58]. Therefore, reduced abundance of SCFA-producing bacteria may contribute to impaired barrier function, a more pro-inflammatory immune phenotype, and disruption of the Treg/Th17 balance, although this mechanism requires validation in kidney transplant cohorts with integrated microbiome, metabolomic, and immunological profiling [59]. Accordingly, SCFA-related pathways should currently be interpreted as mechanistically plausible and potentially relevant, but not as established determinants of graft tolerance or rejection in humans.

Tryptophan-related pathways provide another link between gut microbial metabolism and immune regulation. Microbial metabolism contributes to the generation of several indole derivatives, whereas kynurenine and serotonin-related pathways involve substantial host metabolic regulation; therefore, these pathways should not be considered exclusively bacterial in origin. Immunoregulatory tryptophan-derived metabolites may act partly through the aryl hydrocarbon receptor (AhR), but their effects are ligand- and context-dependent. For example, indole-3-acetic acid and skatole have been shown to exert opposing effects on multidrug resistance protein 1 (MDR1) proteostasis in human colonic epithelial cells, illustrating that tryptophan-derived compounds should not be treated as uniformly beneficial [60]. AhR activation has also been implicated in Th17 and Treg cell differentiation, interleukin 22 (IL-22) production, mucosal homeostasis, epithelial barrier function, and immune regulation [61,62]. In KTR, the direct clinical significance of tryptophan-derived microbial metabolites remains incompletely defined and requires studies integrating diet, renal clearance, immunosuppression, metabolomics, and graft outcomes.

Bile acid metabolism is also shaped by host–microbe interactions. Primary bile acids synthesized in the liver can be transformed by intestinal bacteria into secondary bile acids, which may signal through receptors such as the farnesoid X receptor (FXR) and Takeda G protein-coupled receptor 5 (TGR5), influencing intestinal barrier function, inflammation, and metabolic regulation [63]. Experimental data suggest that selected bile acid metabolites can regulate Th17 and Treg cell differentiation [64]. However, bile acid effects are metabolite-specific and context-dependent, so secondary bile acids should not be interpreted as uniformly protective. In KTR, bile acid signaling may therefore represent another diet–microbiota–host pathway relevant to metabolic and immune regulation [63,64]. Nevertheless, transplant-specific evidence remains limited, and bile acid pathways should be regarded as hypothesis-generating in this setting.

The potential relevance of these mechanisms to kidney transplantation has been discussed by Ardalan et al., who highlighted the gut microbiota as a factor potentially influencing renal transplant outcomes through immune regulation, microbial metabolism, and interactions with immunosuppressive therapy [33]. In this framework, reduced microbial diversity and lower production of microbial metabolites involved in immune and epithelial homeostasis may contribute to a more inflammatory immune profile, whereas dysbiosis may also affect the pharmacokinetics or pharmacodynamics of immunosuppressive drugs [10,35]. Kim et al. further emphasized that dysbiosis in kidney transplantation may be associated with increased susceptibility to opportunistic infections, which can further activate immune pathways and indirectly influence graft-related risk [35]. These interactions illustrate the complexity of the post-transplant ecosystem, in which dysbiosis, infection, immunosuppression, inflammation, and graft function may influence one another bidirectionally [33,35]. Such bidirectional interactions complicate causal interpretation, because microbiota alterations may be both a contributor to and a consequence of post-transplant complications, treatment exposure, or impaired graft function.

Overall, host–microbe interactions provide a biologically plausible link between gut dysbiosis and transplant-related immune regulation. The most relevant pathways include PRR-mediated innate immune activation, Treg/Th17 balance, intestinal barrier integrity, microbial translocation, and immunomodulatory metabolites such as SCFAs, tryptophan-derived compounds, and bile acid derivatives. However, because many mechanistic conclusions are extrapolated from experimental models or non-transplant inflammatory diseases, these pathways should currently be viewed as explanatory mechanisms rather than validated predictors of graft tolerance or transplant outcome [34,35]. This distinction is important because direct KTR evidence remains limited and predominantly observational.

## 5. Post-Transplant Complications and Microbiota-Related Mechanisms

KTRs are particularly susceptible to perturbations of the gut microbiota because they enter transplantation with pre-existing CKD-associated dysbiosis and are subsequently exposed to perioperative stress, hospitalization, broad-spectrum antibiotics, dietary changes, infections, and long-term immunosuppressive therapy [1,25]. These factors may contribute to reduced microbial diversity, depletion of SCFA-producing and other commensal taxa involved in intestinal homeostasis, expansion of opportunistic microorganisms, and altered microbial metabolism. Within this clinical context, gut microbiota alterations have been associated with several post-transplant complications, including gastrointestinal symptoms, diarrhea, infectious complications, UTI, and DGF. Overall, microbiota-related findings in this area should be interpreted as potential contributors or markers of post-transplant vulnerability rather than as established causal determinants of clinical outcomes [25,30,65]. The evidence in this section is derived mainly from small observational KTR cohorts, complemented by studies of non-gut microbial compartments and broader transplant or infection literature. Therefore, each association should be interpreted in relation to antibiotic exposure, immunosuppression, infection, hospitalization, diarrhea, diet, renal function, and time since transplantation.

### 5.1. Gastrointestinal Complications and Diarrhea

Diarrhea is one of the most frequent gastrointestinal complications after kidney transplantation and may substantially affect patient well-being, hydration status, immunosuppressive drug exposure, and healthcare utilization [25]. Its etiology is usually multifactorial and includes infections, antibiotic exposure, mycophenolate-related gastrointestinal toxicity, dietary changes, and post-transplant dysbiosis. Therefore, diarrhea in KTR should not be attributed to gut microbiota disruption alone, but the gut microbiota may represent an important component of the broader pathophysiological context [25,66].

Lee et al. demonstrated that diarrhea in KTR was associated with gut microbiota dysbiosis, including reduced microbial diversity and compositional changes involving taxa potentially relevant to intestinal homeostasis. These findings support the concept that post-transplant diarrhea may be accompanied by microbial ecosystem disruption. Nevertheless, the directionality of this relationship remains difficult to determine, because diarrhea itself, antimicrobial treatment, dietary changes, infections, and adjustments in immunosuppressive therapy can all modify microbiota composition [25]. Thus, this study provides direct clinical evidence of an association between diarrhea and altered gut microbiota in KTR, but it does not establish whether dysbiosis precedes, follows, or amplifies diarrheal episodes.

Microbial metabolism may also be relevant to gastrointestinal complications after transplantation. In advanced kidney disease, gut microbial metabolism contributes to the generation of uremic toxin precursors and other metabolites, including indoxyl sulfate, *p*-cresyl sulfate, and TMAO. Their accumulation depends strongly on renal clearance, hepatic metabolism, diet, and microbial composition. In recipients with delayed or incomplete graft function, persistence of uremia-related metabolic disturbances may contribute to intestinal barrier dysfunction and inflammatory signaling, particularly when graft function remains impaired [16,67,68]. Accordingly, metabolite concentrations in transplant recipients should not be interpreted solely as markers of microbial production, because they may also reflect residual kidney function, renal clearance, dietary intake, host metabolism, and graft injury.

Among gastrointestinal infectious causes, CDI represents a particularly important complication in transplant recipients. KTRs may be vulnerable to *C. difficile* infection because of antibiotic exposure, hospitalization, immunosuppression, and disruption of colonization resistance. *C. difficile* infection may present with persistent diarrhea and can contribute to dehydration, graft dysfunction, and increased morbidity. However, it should be considered one of several possible causes of post-transplant diarrhea rather than the dominant explanation for all gastrointestinal symptoms in this population [26,28]. Because antibiotic exposure is both a risk factor for *C. difficile* infection and a major modifier of gut microbiota composition, it represents a central confounder in studies linking dysbiosis with post-transplant gastrointestinal complications.

### 5.2. Infectious Complications: C. difficile Infection and Urinary Tract Infections

Infections remain one of the major causes of morbidity and mortality after kidney transplantation, reflecting the combined effects of immunosuppression, surgical and urological factors, antimicrobial exposure, and recipient comorbidity [27]. From a gut microbiota perspective, infection risk is relevant because antibiotic therapy and hospitalization can disrupt commensal microbial communities, while dysbiosis may favor expansion of opportunistic or antibiotic-resistant taxa. Nevertheless, infection risk after transplantation is multifactorial, and gut microbiota alterations should be interpreted as one component of a complex post-transplant ecosystem rather than as an isolated cause of infectious complications [1,26].

*C. difficile* infection illustrates how disruption of the intestinal microbial ecosystem can become clinically relevant in immunosuppressed recipients. In KTR, *C. difficile* infection has been associated with adverse clinical outcomes, although these associations may reflect both the infection itself and the broader severity of illness, antibiotic exposure, hospitalization, and comorbidity burden. Recent reviews also discuss fecal microbiota transplantation (FMT) as a potential therapeutic option for recurrent or refractory *C. difficile* infection after transplantation. However, FMT should be framed as an indication-specific strategy for selected infectious complications, not as a general intervention to improve graft outcomes or restore a presumed “normal” microbiota state in KTR [28,29].

Urinary tract infections are among the most common infectious complications after kidney transplantation. Their risk is shaped by multiple factors, including female sex, diabetes, urinary catheters, ureteral stents, urological abnormalities, immunosuppressive intensity, antimicrobial exposure, and prior colonization with resistant organisms [31]. The gut may act as a reservoir for uropathogens, and dysbiosis may favor expansion of opportunistic or antibiotic-resistant taxa that can subsequently colonize the urinary tract. However, this should not be simplified into a direct linear pathway in which intestinal barrier disruption alone causes urinary infection [30,31]. The relationship between gut microbiota composition and UTI risk is likely mediated by colonization resistance, antimicrobial selection pressure, urological factors, host immunity, and prior pathogen exposure.

Moghaddam et al. described gut microbiota alterations in KTR and reported early post-transplant UTI and DGF events in a small prospective cohort. Because of the limited sample size and low number of clinical events, these findings should be considered preliminary and require validation in larger prospective cohorts [30]. This study provides direct KTR evidence, but it does not establish independent predictive value of gut microbiota profiles for UTI or DGF.

The distinction between UTI and asymptomatic bacteriuria is clinically important. Although bacteriuria is common after kidney transplantation, treatment of asymptomatic bacteriuria has not consistently improved graft function or patient prognosis. This observation is relevant to microbiome-oriented care because unnecessary antibiotic exposure may further disrupt commensal communities and promote antimicrobial resistance. Therefore, infection prevention in KTR should combine early recognition of clinically significant infections, management of urological and metabolic risk factors, and antibiotic stewardship rather than indiscriminate eradication of bacterial colonization [31,32]. This also highlights that microbiota-oriented interpretation should not lead to unnecessary antimicrobial interventions in the absence of clear clinical benefit.

### 5.3. Delayed Graft Function and Early Microbial Signatures

DGF is an important early complication after kidney transplantation and is primarily driven by ischemia–reperfusion injury, donor and recipient factors, cold ischemia time, perioperative hemodynamics, inflammation, and early post-transplant injury. Microbiota-related mechanisms may plausibly interact with these pathways through inflammatory signaling, microbial metabolites, oxidative stress, and immune regulation, while early studies suggest that host-associated microbial signatures may reflect early graft vulnerability [30,65]. However, DGF should be considered primarily a graft- and perioperative injury phenotype, and microbial signatures should currently be interpreted as potential correlates or adjunctive biomarkers rather than established causal drivers.

Xiang et al. investigated early salivary microbiota profiles after kidney transplantation and reported that selected microbial signatures were associated with subsequent DGF. Because this study assessed salivary rather than gut microbiota, it should be presented as complementary evidence that host-associated microbial communities across mucosal compartments may reflect early graft vulnerability [65]. It should not be cited as direct evidence for gut microbiota involvement in DGF.

Mechanistically, microbial metabolites and dysbiosis-related inflammatory signals may influence systemic inflammation, endothelial activation, oxidative stress, and immune responses, all of which are relevant to ischemia–reperfusion injury and early graft adaptation. These pathways may interact with established non-microbial determinants of DGF, including clinical severity, antibiotic exposure, perioperative stress, and graft-related injury [17,69]. Taken together, available studies suggest that early microbial signatures may reflect perioperative stress, inflammation, and early graft injury, but their value as independent predictors of DGF remains to be established in larger longitudinal studies. Future studies should integrate gut microbiota profiling, non-gut microbial compartments, metabolomics, perioperative variables, antimicrobial exposure, donor characteristics, ischemia time, and biopsy or clinically adjudicated graft outcomes.

### 5.4. Immunosuppressive Therapy, Antibiotics, and Microbiota Disruption

Post-transplant complications are also shaped by the effects of immunosuppressive and antimicrobial therapies on the intestinal ecosystem. Antibiotics are among the strongest modifiers of gut microbial composition and may reduce colonization resistance, deplete commensal taxa involved in intestinal homeostasis, and favor expansion of opportunistic or resistant organisms [1]. In parallel, immunosuppressive agents may influence the gut microbiota indirectly through immune modulation, gastrointestinal toxicity, metabolic effects, and infection risk. These treatment-related effects are particularly relevant because they can both contribute to dysbiosis and be modified by dysbiosis, creating bidirectional interactions between therapy, microbial ecology, and post-transplant complications [1,25]. For this reason, antibiotic exposure and immunosuppressive regimen should be treated as core confounders in transplant microbiome studies rather than as background clinical details.

Mycophenolate mofetil and mycophenolic acid are particularly relevant in the context of gastrointestinal symptoms. Mycophenolate-related enteropathy and diarrhea are recognized clinical problems after kidney transplantation, and experimental data suggest that mycophenolate-induced intestinal injury may be accompanied by alterations in gut microbiota composition and SCFA profiles. However, most mechanistic data remain preclinical, and the clinical significance of mycophenolate-associated microbiota changes in KTR requires further investigation [66]. In clinical cohorts, mycophenolate exposure may confound associations between diarrhea, microbiota composition, and graft-related outcomes because it can contribute to gastrointestinal symptoms while also reflecting immunosuppressive intensity.

Although immunosuppressive regimens may influence gut microbial composition, dose adjustment should remain guided by established clinical indications, rejection risk, infection risk, therapeutic drug monitoring, adverse effects, and graft function rather than by microbiome effects alone. At present, microbiome-informed modification of immunosuppression is not a validated clinical strategy. Future studies should determine whether microbiota-related markers can help identify patients at risk of gastrointestinal toxicity, infectious complications, or unstable immunosuppressive drug exposure, but such approaches remain investigational [25,66]. This distinction is clinically important because microbiome associations should not be translated prematurely into changes in immunosuppressive management.

Overall, post-transplant complications associated with gut microbiota disruption are likely to arise through several overlapping mechanisms, including impaired colonization resistance, altered microbial metabolism, intestinal barrier dysfunction, microbial translocation, chronic low-grade inflammation, and treatment-related ecological pressure. These mechanisms provide a plausible framework linking dysbiosis with diarrhea, *C. difficile* infection, UTIs, and DGF. However, the available evidence remains largely associative, often based on small cohorts, heterogeneous sampling strategies, and incomplete adjustment for antibiotic exposure, immunosuppressive therapy, diet, renal function, diarrhea, infection, hospitalization, time since transplantation, and comorbidity. Therefore, microbiota-related signatures should currently be considered promising but investigational markers of post-transplant complications, and prospective studies integrating microbiome profiling, metabolomics, antimicrobial exposure, immunological markers, and clinically adjudicated outcomes are needed before microbiota-targeted strategies can be incorporated into routine transplant care [26,30,65]. The main post-transplant complications discussed in this section and their microbiota-related associations are summarized in Table 2. These studies illustrate potential links between microbial dysbiosis, gastrointestinal complications, infectious outcomes, UTIs, and DGF, although most findings remain preliminary and should be interpreted as associative rather than causal.

### 5.5. Integrated Conceptual Framework of Microbiome-Related Post-Transplant Complications

**Figure 3:**
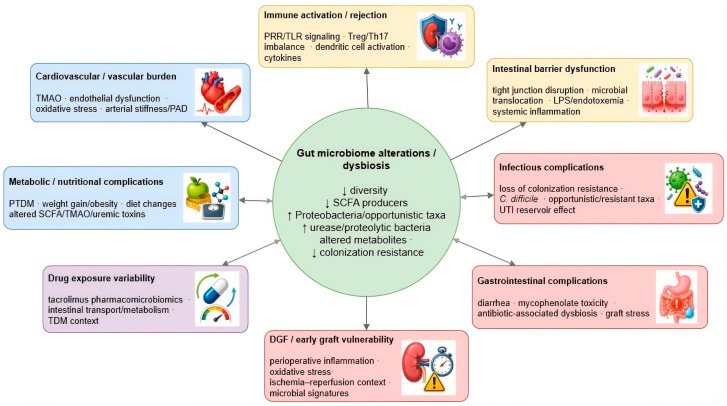
Gut microbiome alterations as a potential modifier of post-transplant complications and graft-related risk in KTR. Post-transplant dysbiosis may include reduced microbial diversity, depletion of short-chain fatty acid-producing taxa, expansion of Proteobacteria or opportunistic taxa, altered microbial metabolite production, and impaired colonization resistance. These alterations may interact with intestinal barrier dysfunction, microbial translocation, immune activation, Treg/Th17 imbalance, infectious and gastrointestinal complications, early graft vulnerability, immunosuppressive drug exposure variability, metabolic disturbances, and cardiovascular or vascular burden. Arrows indicate proposed links; bidirectional arrows indicate potential feedback loops. Colors indicate conceptual domains: yellow—mechanistic pathways; red—clinical post-transplant complications; blue—metabolic, nutritional, cardiovascular, and vascular consequences; purple—immunosuppressive drug exposure variability. The figure is a conceptual framework based on associative and mechanistic evidence rather than established causality. Abbreviations: DGF—delayed graft function; KTR—kidney transplant recipients; LPS—lipopolysaccharide; PAD—peripheral arterial disease; PRR—pattern-recognition receptor; PTDM—post-transplant diabetes mellitus; SCFA—short-chain fatty acid; TDM—therapeutic drug monitoring; TLR—Toll-like receptor; TMAO—trimethylamine *N*-oxide; Treg—regulatory T cell; UTI—urinary tract infection.

Beyond individual post-transplant complications, gut microbiome alterations may interact with overlapping gastrointestinal, infectious, immunological, metabolic, graft-related, and vascular pathways. To improve readability and integrate these mechanisms across the manuscript, Figure 3 summarizes the proposed conceptual links between dysbiosis, post-transplant complications, and potential mechanisms of graft injury. Because these links are supported mainly by associative clinical studies and mechanistic plausibility, the framework should be interpreted as hypothesis-generating rather than causal.

## 6. Cardiovascular and Vascular Consequences After Kidney Transplantation

Although kidney transplantation improves survival and reduces cardiovascular risk compared with continued dialysis, cardiovascular disease remains a major cause of post-transplant morbidity and mortality [70,71,72,73]. In the context of this review, the key issue is not general vascular injury after transplantation, but whether gut dysbiosis and microbiota-associated metabolites are linked to persistent endothelial dysfunction and vascular risk. Endothelial abnormalities in KTR may be shaped by oxidative stress, impaired nitric oxide bioavailability, chronic inflammation, residual kidney dysfunction, calcineurin inhibitor exposure, and incomplete recovery from the uremic milieu [2,74,75,76,77]. These mechanisms may overlap with dysbiosis-related pathways, including impaired intestinal barrier integrity, microbial translocation, microbiota-derived metabolites, and chronic low-grade inflammation [78]. However, direct evidence linking gut microbiota composition to cardiovascular outcomes in KTR remains limited, and most mechanistic interpretation is extrapolated from CKD, cardiovascular, or experimental literature.

**Figure 4:**
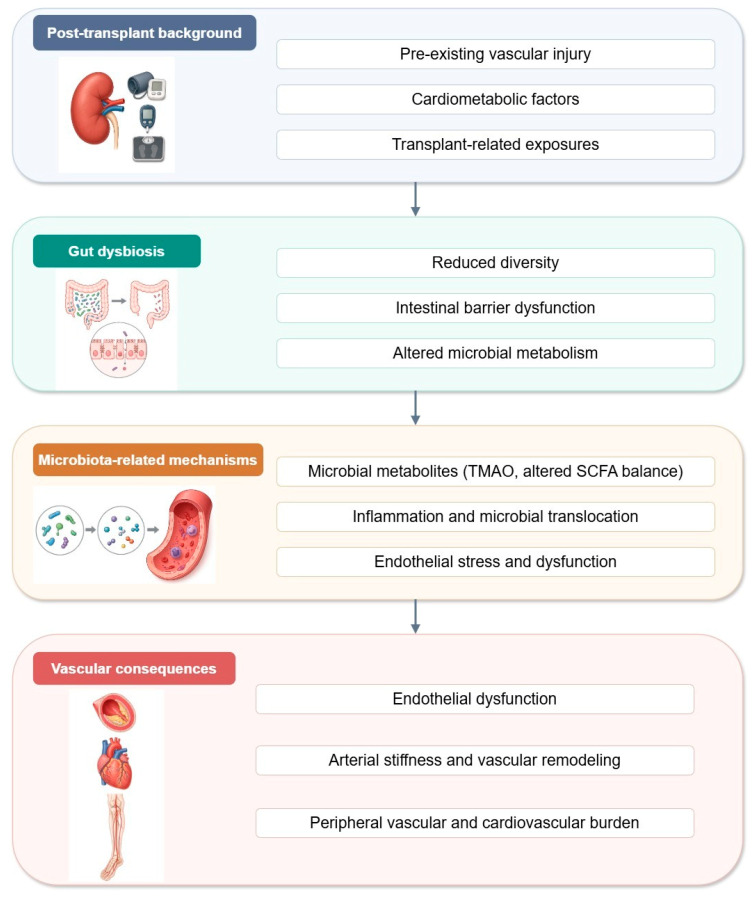
Proposed links between post-transplant dysbiosis and persistent cardiovascular and vascular risk after kidney transplantation. Post-transplant gut dysbiosis may modulate vascular risk through reduced microbial diversity, intestinal barrier dysfunction, altered microbial metabolism, microbiota-derived metabolites such as trimethylamine *N*-oxide, inflammation and microbial translocation, and endothelial stress. These pathways may be associated with or plausibly contribute to endothelial dysfunction, arterial stiffness and vascular remodeling, peripheral arterial disease, and persistent cardiovascular burden despite improved renal function after transplantation. Blue boxes indicate post-transplant background factors, green boxes represent gut dysbiosis-related alterations, orange boxes denote microbiota-related mechanisms, and pink boxes show cardiovascular and vascular consequences. The figure represents a conceptual framework based on associative and mechanistic evidence rather than established causality. Abbreviations: SCFA—short-chain fatty acid; TMAO—trimethylamine *N*-oxide.

Within the microbiota-oriented framework of this review, post-transplant dysbiosis may be associated with endothelial stress through gut-derived inflammatory and metabolic signaling. Reduced microbial diversity, impaired barrier function, increased exposure to microbial products, and altered metabolite production may contribute to endothelial activation, oxidative stress, and atherothrombotic pathways. However, the available evidence remains largely associative, and the gut microbiota should be interpreted as a potential modifier of post-transplant vascular risk rather than as an isolated determinant of cardiovascular disease [1,2,5]. This distinction is important because vascular risk after transplantation is strongly influenced by age, diabetes, hypertension, dyslipidemia, residual graft dysfunction, immunosuppressive therapy, inflammation, prior dialysis exposure, and lifestyle-related factors. A conceptual overview of the proposed links between post-transplant dysbiosis, microbiota-related signaling, and persistent cardiovascular and vascular burden is presented in Figure 4.

Among microbiota-derived compounds, TMAO currently represents the most direct link between gut microbial metabolism and vascular risk in KTR. In the broader cardiovascular and CKD literature, elevated TMAO concentrations have been associated with endothelial dysfunction, vascular inflammation, platelet activation, and atherosclerotic progression. Transplant-specific evidence also suggests a similar association after kidney transplantation, as higher serum TMAO levels were independently associated with PAD, defined by a reduced ankle–brachial index. These findings support the concept that gut-derived metabolites may serve as markers of an unfavorable vascular milieu and highlight the need to consider diet, hepatic metabolism, and residual graft function when interpreting circulating TMAO concentrations [6,70,71]. Importantly, circulating TMAO should not be interpreted as a direct measure of microbial activity alone, because its concentration is influenced by dietary intake of choline- and carnitine-containing foods, gut microbial conversion, hepatic oxidation, renal clearance, graft function, and comorbidity burden. Therefore, the association between TMAO and vascular outcomes in KTR should be interpreted as biomarker evidence rather than proof of causality.

The broader vascular phenotype after transplantation also includes increased arterial stiffness, which may persist despite successful graft implantation and may have prognostic significance. Although arterial stiffness often improves after transplantation compared with dialysis, this improvement is incomplete and does not necessarily normalize vascular risk. Higher aortic stiffness has been linked to mortality prediction in KTR, while smaller clinical studies suggest that arterial stiffening may coexist with persistent endothelial abnormalities and adverse blood pressure patterns [76,79,80]. In this context, arterial stiffness may be viewed as one clinical manifestation of persistent vascular injury potentially influenced by metabolic, inflammatory, and microbiota-related mechanisms. However, available transplant-specific data do not yet establish whether microbiota-related metabolites improve vascular risk prediction beyond established clinical and hemodynamic markers.

Markers of endothelial and vascular injury may complement microbiome-related risk assessment. Circulating endostatin has been reported as an independent predictor of graft loss after kidney transplantation, suggesting that vascular and endothelial pathways contribute to long-term graft prognosis [81]. Future studies should therefore assess whether microbiota-derived metabolites improve prediction beyond established vascular, inflammatory, and graft-function biomarkers. This approach would allow microbiota-associated metabolites to be evaluated within a broader vascular biomarker framework rather than in isolation.

Lifestyle-related exposures, including diet and physical activity, should be considered in future studies because they may influence both microbiome composition and vascular-inflammatory phenotypes in KTR [82]. Nevertheless, the current evidence base remains limited by relatively small transplant-specific cohorts, cross-sectional designs, and incomplete integration of microbiome, metabolomic, and vascular phenotyping data. Therefore, microbiota-associated cardiovascular signals should presently be regarded as hypothesis-generating and mechanistically informative rather than definitive proof of causality [72,83,84]. Overall, microbiota-derived metabolites, particularly TMAO, provide a plausible link between diet, gut microbial metabolism, endothelial dysfunction, and persistent cardiovascular risk after kidney transplantation; however, their independent prognostic and therapeutic value requires further validation. Future studies should integrate gut microbiota profiling, metabolomics, dietary assessment, graft function, renal clearance, endothelial biomarkers, vascular imaging, and adjudicated cardiovascular outcomes to determine whether microbiota-related markers add clinically meaningful information beyond established cardiovascular and transplant-related risk factors.

## 7. Graft Function and Outcomes

### 7.1. Rejection-Related Microbiome Alterations and Candidate Early Markers

Emerging evidence suggests that KTRs who develop graft rejection exhibit gut microbial profiles that differ from those observed in recipients with stable graft function. Across currently available studies, rejection has generally been associated with reduced microbial diversity and compositional shifts involving taxa linked to SCFA production and intestinal metabolic homeostasis. However, these findings should be interpreted cautiously, as the evidence remains based predominantly on observational studies with limited sample size and variable study design [7,85,86]. In addition, rejection itself, intensification of immunosuppression, antibiotic exposure, infection, diarrhea, reduced graft function, and changes in diet or hospitalization status may all influence gut microbiota composition. Therefore, rejection-associated microbial signatures should currently be interpreted as candidate biomarkers or correlates rather than established mediators of alloimmune injury.

Of particular interest, Holle et al. reported that alterations in gut microbiome composition were detectable before clinically overt rejection, suggesting that microbiome changes may precede graft instability in at least a subset of recipients. This observation supports the concept that gut microbial shifts may represent candidate early markers of an evolving adverse graft course. At present, however, it remains unclear whether these changes contribute directly to rejection biology or instead reflect concurrent alterations in inflammation, renal function, diet, antimicrobial exposure, or immunosuppressive treatment [7,86]. Accordingly, the temporal relationship observed in this study strengthens the hypothesis-generating value of microbiome profiling, but does not establish causality or clinical utility without external validation.

The available evidence is somewhat more consistent in studies focusing on antibody-mediated rejection (AMR), although it should still be regarded as preliminary. Wang et al. demonstrated that recipients with AMR had significantly altered gut microbial composition compared with stable transplant recipients, including reduced richness and differences in the abundance of selected taxa. Complementary metabolomic data further suggested that AMR is accompanied by changes in intestinal metabolic profiles, indicating that rejection-associated microbial perturbations may involve both taxonomic and functional alterations. Nevertheless, these findings require validation in larger longitudinal cohorts with standardized phenotyping and biopsy-correlated outcome assessment before they can be considered robust rejection biomarkers [85,87]. Such studies should also account for treatment changes, infection, antibiotic exposure, renal function, proteinuria, diet, and time after transplantation, because these factors may confound associations between microbial profiles and rejection status.

### 7.2. Microbial Correlates of Graft Function and Chronic Allograft Injury

Beyond rejection episodes, microbial features have also been associated with quantitative measures of graft performance. In a study of living donor kidney transplantation, Kim et al. showed that greater similarity of gut microbiota composition between donor and recipient was associated with better early allograft function, particularly at 6 months after transplantation. These findings suggest that the microbiome may be relevant not only to immune activation, but also to early post-transplant graft adaptation. This association may reflect both transplant-related host–microbe interactions and shared environmental or dietary exposures between donors and recipients [88]. Because the study was conducted in the setting of living donor transplantation, generalizability to deceased donor transplantation and to recipients with more complex perioperative courses requires further investigation.

Data directly linking gut microbiota to chronic allograft dysfunction remain limited. However, the broader literature suggests that microbial ecosystem disturbances may accompany progressive graft injury. In this context, evidence from urinary microbiome studies may be considered complementary, although it falls outside the strict scope of gut microbiota. Specifically, alterations in urinary microbial composition have been associated with chronic allograft dysfunction and with interstitial fibrosis and tubular atrophy detected in surveillance biopsies. Although these findings cannot be directly extrapolated to the gut microbiota, they support the broader concept that host–microbe disturbances across biological compartments may accompany chronic graft injury [89,90]. These studies should therefore be described as complementary non-gut microbiome evidence, not as direct evidence that gut dysbiosis contributes to chronic allograft injury.

From a mechanistic perspective, these associations are biologically plausible because persistent dysbiosis may contribute to graft dysfunction through impaired barrier integrity, altered metabolite production, and sustained low-grade inflammatory signaling. Conversely, microbial alterations may also emerge secondarily in recipients with poorer graft function, greater comorbidity burden, repeated antibiotic exposure, or more complicated post-transplant courses. Therefore, microbiome-related changes should currently be interpreted as candidate correlates of graft dysfunction rather than established mediators of chronic allograft injury [7,88]. This bidirectionality is particularly important in transplant recipients, because declining graft function may itself modify circulating metabolite concentrations, dietary restrictions, medication exposure, inflammation, and the intestinal ecosystem.

Biomarker studies in KTR further illustrate the importance of accounting for graft function when interpreting circulating metabolites. Higher concentrations of intact and *C*-terminal fibroblast growth factor 23 (FGF23) have been associated with graft survival, indicating that mineral-metabolism biomarkers may provide prognostic information beyond conventional measures of kidney function [91]. Such findings support the integration of microbiome-derived metabolites with established metabolic and graft-related biomarkers rather than evaluating them in isolation.

### 7.3. Microbiota-Derived Metabolites, Graft Failure, and Long-Term Outcomes

Among microbiota-related markers, TMAO currently represents one of the most clinically relevant candidates in relation to long-term transplant outcomes. In renal transplant recipients, higher circulating TMAO concentrations and their dietary determinants were associated with an increased future risk of graft failure. More recent data also suggest an association between higher post-transplant plasma TMAO levels and all-cause mortality. These findings support the potential prognostic relevance of microbiota-derived metabolites after kidney transplantation and highlight the need to interpret circulating TMAO as a diet–microbiota–host co-metabolite influenced by gut microbial metabolism, dietary precursors, hepatic metabolism, renal clearance, and baseline graft function [92,93]. Therefore, elevated TMAO should not be interpreted as evidence that microbial production alone drives graft failure or mortality; it may also reflect impaired clearance, dietary exposure, comorbidity burden, systemic inflammation, and residual graft dysfunction.

Additional evidence suggests that other microbiota-related metabolites may also carry prognostic information. Korytowska et al. reported that salivary indoxyl sulfate, particularly when assessed together with proteinuria, may help identify recipients at risk of graft deterioration. Although this approach does not directly characterize gut microbial composition, it remains relevant within a microbiome-oriented framework because indoxyl sulfate is generated through host–microbe metabolic interactions. Thus, microbiota-derived uremic toxins may reflect biological processes linked to ongoing graft injury and may complement established markers such as proteinuria in risk stratification [94]. However, salivary indoxyl sulfate should be considered complementary biomarker evidence rather than direct gut microbiota evidence. Its interpretation also requires consideration of renal clearance, proteinuria, diet, host metabolism, and graft function.

A further indirect pathway linking the microbiome to graft outcomes may involve tacrolimus pharmacokinetics. Recent studies suggest that gut microbiota and microbiota-related metabolites may contribute to intra-patient variability of tacrolimus exposure, and such variability has been associated with less favorable post-transplant outcomes. Although microbiome-dependent pharmacokinetic variation should be distinguished from rejection biology, these findings support the view that microbial factors may influence graft trajectory through effects on immunosuppressive drug handling [95,96]. At the same time, tacrolimus variability is multifactorial and may be influenced by adherence, cytochrome P450 3A5 (CYP3A5) genotype, liver function, drug interactions, diarrhea, dose changes, time after transplantation, and graft function. Microbiome-informed tacrolimus management therefore remains investigational and should not replace established therapeutic drug monitoring.

Overall, current evidence supports associations between microbial and metabolite-related alterations and clinically relevant transplant endpoints, including rejection, early graft function, chronic graft injury, graft deterioration, graft failure, and mortality. Because available studies differ in population, biospecimen type, analytical methodology, and endpoint definition, microbiome- and metabolite-related signals should currently be regarded as promising investigational markers rather than validated clinical tools. Further longitudinal studies integrating serial microbiome profiling with metabolomics, drug exposure data, biopsy findings, and hard graft endpoints are needed to determine which signals are reproducible and clinically actionable [7,86,88,92]. Future studies should also incorporate established graft-related, metabolic, vascular, and nutritional biomarkers to determine whether microbiota-derived metabolites add prognostic value beyond conventional clinical predictors. Overall, microbiota-derived metabolites should be evaluated within a broader biomarker framework that includes renal clearance, nutritional status, mineral metabolism, endothelial injury, and graft function. Multimarker and multi-omics approaches may be more informative than isolated microbial or metabolite measurements.

## 8. Nutritional Modulation of the Gut Microbiota After Kidney Transplantation

### 8.1. Dietary Fiber, SCFA Production, and Barrier–Immune Homeostasis

Dietary fiber represents one of the most important nutritional determinants of gut microbial metabolic activity after kidney transplantation. In contrast to the pre-transplant period, when patients with advanced CKD or dialysis-dependent kidney failure are frequently exposed to dietary restrictions, successful transplantation may be followed by relaxation of dietary limitations, improved appetite, and broader food choices. These changes may modify the availability of fermentable substrates for intestinal bacteria and thereby influence the production of SCFAs, including acetate, propionate, and butyrate. SCFAs are generated mainly through bacterial fermentation of non-digestible carbohydrates and resistant starches, and they represent a major link between diet, microbial activity, intestinal barrier function, and systemic immune regulation [97]. However, direct clinical evidence that increasing dietary fiber modifies graft outcomes through SCFA-mediated mechanisms in KTR remains limited.

From a microbiological perspective, higher availability of fermentable fiber may support bacterial taxa involved in SCFA production, including *Faecalibacterium*, *Roseburia*, and members of the Lachnospiraceae and Ruminococcaceae families. This is relevant in KTR because depletion of SCFA-producing taxa has been described as part of the dysbiosis phenotype observed in CKD and after transplantation. Reduced SCFA production may contribute to impaired epithelial barrier function, increased permeability to microbial products, and a more pro-inflammatory intestinal environment. Conversely, butyrate supports colonocyte metabolism and tight junction integrity, whereas acetate and propionate may contribute to systemic metabolic and immune signaling [24]. These pathways are biologically plausible, but their clinical relevance after transplantation should be interpreted in the context of graft function, diet, antibiotic exposure, diarrhea, and immunosuppressive therapy.

The immunological relevance of SCFAs is particularly important in transplantation because these metabolites may influence regulatory and effector immune responses. SCFAs can signal through GPR41, GPR43, and GPR109A, and may also modulate gene expression through HDAC inhibition. Through these mechanisms, SCFAs may support Treg differentiation, reduce pro-inflammatory cytokine production, and contribute to barrier–immune homeostasis. Experimental kidney transplant data suggest that a high-fiber diet or acetate supplementation may promote donor-specific tolerance through induction of Tregs, providing mechanistic support for a diet–microbiota–SCFA–immune regulation axis in transplantation [34]. However, these findings should be interpreted as mechanistic evidence rather than as a direct clinical recommendation for unrestricted high-fiber supplementation in all KTR. At present, there is insufficient transplant-specific clinical evidence to conclude that fiber-induced SCFA changes improve rejection risk, graft survival, or long-term patient outcomes.

In clinical practice, fiber intake after kidney transplantation should be individualized. A diet richer in vegetables, fruits, legumes, whole grains, and other fiber-containing foods may be beneficial for microbial diversity, SCFA production, bowel function, and cardiometabolic health. Nevertheless, KTRs differ substantially in graft function, serum potassium and phosphorus levels, gastrointestinal tolerance, immunosuppressive regimen, antibiotic exposure, and risk of diarrhea or infection. Therefore, dietary fiber should be increased gradually and adjusted to graft function, metabolic profile, and gastrointestinal symptoms. In recipients with stable graft function and good gastrointestinal tolerance, higher fiber intake may represent a rational nutritional strategy to support SCFA-producing microbial communities. In contrast, in patients with impaired graft function, hyperkalemia, severe diarrhea, or active gastrointestinal complications, fiber recommendations may require closer clinical and dietetic supervision [97,98]. This individualized approach is particularly important because dietary changes may influence both microbiota composition and clinical outcomes, making diet a potential intervention and a major confounder in observational microbiome studies.

Overall, dietary fiber is a modifiable nutritional factor that may influence gut microbiota composition and SCFA-mediated barrier–immune homeostasis after kidney transplantation. Its potential relevance extends beyond gastrointestinal health and may include immune regulation, metabolic risk, and cardiovascular protection. However, transplant-specific clinical evidence remains limited, and future studies should integrate detailed dietary assessment with microbiome profiling, SCFA measurement, immunological markers, graft function, and patient-centered outcomes [98]. Such studies should distinguish mechanistic SCFA-related effects from broader benefits of cardiometabolically favorable dietary patterns.

### 8.2. Protein Source, Choline, L-Carnitine, and Microbiota-Derived Uremic and Cardiovascular Toxins

Protein intake after kidney transplantation requires careful interpretation because nutritional needs change across the transplant timeline. In the early post-transplant period, adequate protein intake is important for wound healing, recovery from surgery, preservation of lean body mass, and counteracting the catabolic effects of corticosteroids and acute illness. During long-term follow-up, however, protein recommendations should be individualized according to graft function, metabolic status, body composition, cardiovascular risk, and comorbidities. Therefore, protein intake in KTR should not be considered only in quantitative terms, but also in relation to protein source and its potential effects on gut microbial metabolism [99].

From a microbiota perspective, diets with a higher proportion of animal protein and lower availability of fermentable carbohydrates may favor proteolytic fermentation in the colon. This process can increase the generation of microbial precursors of uremic toxins, including indole and *p*-cresol, which are subsequently converted by host metabolism into indoxyl sulfate and *p*-cresyl sulfate. These metabolites have been linked to inflammation, oxidative stress, endothelial dysfunction, and progression of kidney and cardiovascular disease, particularly when renal clearance is reduced. In KTR, their concentrations may therefore reflect the combined influence of protein source, microbial metabolism, hepatic processing, residual or graft kidney function, and medication-related factors [16,68]. Accordingly, these metabolites should be interpreted as host–microbe co-metabolites rather than as direct markers of gut microbial activity alone.

The clinical implication is not that protein should be uniformly restricted after kidney transplantation, but that the quality, timing, and context of protein intake should be considered. In stable KTR, a dietary pattern that avoids excessive animal protein intake while ensuring adequate total protein may help reduce the substrate burden for proteolytic fermentation without compromising nutritional status. This is particularly relevant in recipients with impaired graft function, persistent inflammation, cardiovascular disease, obesity, or post-transplant diabetes mellitus (PTDM). Conversely, overly restrictive protein intake may be harmful in recipients with malnutrition, sarcopenia, frailty, poor wound healing, or recent surgery. Thus, protein guidance should balance graft function, metabolic risk, and preservation of muscle mass rather than rely on a single microbiome-focused target [99].

Choline- and L-carnitine-rich foods represent another important diet–microbiota–host pathway because they provide substrates for microbial TMA generation and subsequent hepatic conversion to TMAO. Red meat is a major dietary source of L-carnitine, whereas eggs, meat, fish, and some dairy products contribute dietary choline. In controlled dietary intervention studies, chronic red meat intake increased circulating TMAO concentrations compared with white meat or non-meat protein sources, mainly through increased precursor availability, enhanced microbial TMA/TMAO generation, and altered renal excretion. These findings are particularly relevant to KTR because circulating TMAO concentrations are influenced not only by diet and microbiota, but also by graft function and renal clearance [100,101]. Because the controlled dietary intervention evidence was not generated in KTR, it should be interpreted as supportive mechanistic and nutritional evidence rather than direct transplant evidence.

TMAO is relevant in kidney transplantation because it may connect dietary exposures with cardiovascular and graft-related risk. In CKD populations, higher circulating TMAO concentrations have been associated with lower kidney function, and meta-analytic evidence supports a negative association between TMAO and kidney function. In KTR elevated TMAO has also been linked with adverse graft-related outcomes and vascular risk, including associations with graft failure and PAD. These associations support the biological plausibility of TMAO as a diet–microbiota–host co-metabolite involved in post-transplant risk, although interpretation requires attention to confounding by diet, renal clearance, hepatic metabolism, comorbidity, and inflammation [92,102]. Therefore, elevated TMAO should be considered a candidate biomarker within a broader metabolic and graft-function framework rather than proof that TMAO causally drives graft loss or vascular disease.

From a practical perspective, the aim should be to avoid excessive red meat and highly animal-protein-dominant dietary patterns rather than to eliminate all choline- or carnitine-containing foods. Choline is an essential nutrient, and protein adequacy remains important in KTR; therefore, overly restrictive approaches may create nutritional risks. A balanced dietary pattern that includes adequate protein, greater use of plant-forward protein sources when clinically appropriate, sufficient fiber intake, and moderation of red and processed meat may reduce exposure to microbiota-derived uremic and cardiovascular toxins while preserving nutritional adequacy. This strategy should be individualized according to eGFR, serum potassium and phosphorus, nutritional status, PTDM, gastrointestinal tolerance, cardiovascular risk, and immunosuppressive regimen [99,101]. Such dietary recommendations should be framed primarily as cardiometabolic and nutritional strategies with plausible microbiota-related effects, not as validated microbiome-targeted therapies.

Dietary composition may also influence other microbiota-dependent metabolite pathways relevant to post-transplant immune and metabolic regulation. Diets low in fermentable fiber and high in animal protein may shift microbial metabolism toward proteolytic fermentation, thereby affecting not only indoxyl sulfate and *p*-cresyl sulfate precursors, but also tryptophan-derived indole metabolites. These compounds may interact with AhR-related pathways involved in epithelial barrier function and T-cell regulation, although their effects are ligand-specific and context-dependent. In parallel, dietary fat quality, fiber intake, and gut microbial composition may influence bile acid transformation and signaling through the FXR and TGR5, linking nutrition with intestinal barrier integrity, inflammation, glucose metabolism, and immune regulation. Therefore, tryptophan-derived metabolites and bile acid derivatives should be considered part of a broader diet–microbiota–host metabolic network rather than isolated microbial products [60,63,64]. Most of this evidence remains mechanistic or extrapolated from non-transplant settings and requires validation in kidney transplant cohorts.

### 8.3. Mediterranean-Style and Plant-Forward Dietary Patterns, Weight Gain, and PTDM

Beyond single nutrients, overall dietary patterns may be more clinically relevant for long-term nutritional management after kidney transplantation. Mediterranean-style and plant-forward dietary patterns are of particular interest because they combine higher intake of fiber-rich foods, vegetables, fruits, legumes, nuts, whole grains, and unsaturated fats with lower intake of red and processed meat. This dietary profile may support microbial diversity, increase fermentable substrate availability, reduce excessive exposure to animal protein-derived microbial metabolites, and improve cardiometabolic risk factors. In KTR, these effects are clinically relevant because long-term outcomes are influenced not only by alloimmune injury, but also by hypertension, dyslipidemia, obesity, PTDM, and persistent cardiovascular risk [103]. However, dietary pattern studies should be interpreted carefully because diet may influence outcomes through multiple pathways independent of the gut microbiota.

Among available dietary patterns, the Mediterranean-style diet has the strongest transplant-specific observational support. In a large cohort of KTR, higher adherence to a Mediterranean-style diet was associated with better kidney function outcomes and lower risk of kidney function loss. This association is biologically plausible because this dietary pattern may improve blood pressure, lipid profile, insulin sensitivity, oxidative stress, and inflammatory status, while also providing fiber and polyphenol substrates that may influence gut microbiota composition and microbial metabolite production. However, these findings should be interpreted as supportive evidence for a cardiometabolically favorable dietary framework rather than as proof that Mediterranean-style eating directly modifies graft outcomes through the microbiota [104]. Residual confounding by lifestyle, socioeconomic factors, medication adherence, baseline graft function, comorbidity burden, and overall health behavior remains possible.

Mediterranean-style dietary patterns may also be relevant to PTDM. PTDM is a frequent metabolic complication after kidney transplantation and is influenced by recipient factors, corticosteroids, calcineurin inhibitors, weight gain, inflammation, and lifestyle-related exposures. In KTR, greater adherence to a Mediterranean-style diet has been associated with a lower risk of new-onset diabetes after transplantation. This may reflect the combined effects of higher intake of minimally processed plant foods, lower glycemic burden, improved fat quality, lower red meat exposure, and favorable effects on weight control and insulin sensitivity. Because PTDM increases cardiovascular risk and may adversely affect long-term graft and patient outcomes, dietary strategies that improve glucose metabolism are particularly important in post-transplant care [105]. These data provide direct transplant-specific nutritional evidence, but not direct evidence that microbiota changes mediate the lower PTDM risk.

Plant-forward dietary patterns may provide additional microbiota-related advantages by increasing fermentable carbohydrates and polyphenol exposure while reducing excessive animal protein, red meat, and saturated fat intake. Such patterns may promote SCFA production and may reduce substrate availability for TMAO and selected uremic toxin pathways. Nevertheless, plant-based dietary advice in KTR must be individualized rather than applied uniformly. Recipients with impaired graft function, hyperkalemia, hyperphosphatemia, malnutrition, sarcopenia, gastrointestinal intolerance, or recent surgery may require modified recommendations regarding legumes, nuts, whole grains, fruits, vegetables, and total protein intake. Therefore, a plant-forward approach should be framed as a flexible pattern emphasizing minimally processed plant foods and cardiometabolic quality, not as a strict or unsupervised plant-only diet [103,106]. This distinction is important because nutritional adequacy and electrolyte safety may outweigh theoretical microbiota-related benefits in selected recipients.

Post-transplant weight gain is another major nutritional challenge that may interact with gut microbiota and metabolic risk. After transplantation, improved appetite, reduced uremic symptoms, relaxation of dietary restrictions, corticosteroid exposure, reduced physical activity, and psychological factors may contribute to increased energy intake and fat mass accumulation. Weight gain may then promote insulin resistance, dyslipidemia, hypertension, systemic inflammation, and PTDM, all of which may contribute to persistent cardiovascular risk after transplantation. Because diet also modifies gut microbiota composition, energy balance and dietary quality should be considered together rather than separately in long-term nutritional care of KTR [106,107]. At the same time, weight gain may confound microbiome studies because it can both alter microbial ecology and influence metabolic or graft-related outcomes.

Nutritional biomarkers may also be relevant to long-term graft outcomes. In stable KTR, lower 25-hydroxyvitamin D concentrations, but not 1,25-dihydroxyvitamin D concentrations, were independently associated with graft loss [108]. This finding highlights the need to distinguish dietary or nutritional exposure markers from biologically active metabolites and to consider kidney function, supplementation, inflammation, and immunosuppressive treatment when evaluating nutritional–microbiome interactions. It also supports a broader biomarker framework in which microbiota-derived metabolites are interpreted alongside nutritional, mineral-metabolism, vascular, and graft-function markers.

Overall, Mediterranean-style and plant-forward dietary patterns may represent practical frameworks for aligning microbiota-related mechanisms with established post-transplant nutritional goals. Their potential benefits include higher fiber intake, increased SCFA-generating substrates, lower red and processed meat burden, improved cardiometabolic profile, and better long-term dietary quality. However, their implementation in KTR should be individualized according to eGFR, serum potassium and phosphorus, protein requirements, nutritional status, body composition, PTDM risk, gastrointestinal symptoms, and immunosuppressive regimen. Future studies should combine dietary pattern assessment with microbiome and metabolomic profiling to determine whether these dietary approaches modify microbial pathways in ways that translate into improved patient- and graft-centered outcomes [103,104]. Available evidence on nutritional modulation of the gut microbiota after kidney transplantation includes experimental models, transplant-specific cohort studies, dietary intervention studies, pilot interventional trials, and systematic reviews. Original studies and systematic evidence most directly informing diet–microbiota–metabolite pathways and microbiota-targeted nutritional interventions in this context are summarized in Table 3. The table distinguishes direct transplant-specific evidence from supportive experimental, non-transplant, or broader solid organ transplant evidence.

### 8.4. Probiotics, Prebiotics, Synbiotics, and Individualized Nutrition in Immunosuppressed Recipients

Probiotics, prebiotics, and synbiotics represent potential microbiota-directed adjuncts to nutritional care after kidney transplantation. Their rationale is based on the possibility of modulating gut microbial composition and metabolic activity, supporting SCFA-related pathways, reducing selected uremic toxin pathways, improving bowel symptoms, and restoring colonization resistance after antibiotic exposure. In KTR, these interventions are attractive because they are less invasive than FMT and could theoretically be integrated into dietary management. However, their effects are strain-specific, substrate-specific, dose-dependent, and strongly influenced by baseline dysbiosis, antibiotic exposure, immunosuppressive therapy, gastrointestinal symptoms, and graft function [109]. Therefore, they should not be discussed as general microbiota-restorative therapies, and their use should be evaluated according to indication, safety, and clinical context.

Evidence in KTR remains limited but clinically relevant. In a pilot interventional study, short-course synbiotic treatment reduced plasma *p*-cresol concentrations in KTR, suggesting that modulation of gut microbial metabolism may influence selected uremic toxin-related pathways after transplantation. This finding is important because *p*-cresol and related metabolites reflect proteolytic fermentation and have been associated with inflammatory and cardiovascular pathways in kidney disease. Nevertheless, this study should be interpreted as proof-of-concept rather than evidence that synbiotics improve graft survival, cardiovascular outcomes, or long-term patient prognosis in KTRs [110]. In addition, *p*-cresol concentrations are influenced by diet, microbial metabolism, host processing, and kidney function, so biomarker changes should not be equated with clinical benefit.

Safety is a central consideration because KTRs are chronically immunosuppressed and frequently exposed to antibiotics, antiviral prophylaxis, hospitalization, gastrointestinal complications, and fluctuating graft function. A Cochrane review of synbiotics, prebiotics, and probiotics in SOT recipients found insufficient evidence to support or refute their routine use, highlighting the limited number of adequately designed studies and the need for better assessment of both efficacy and harms. More recent kidney transplant data also suggest that probiotic exposure should not be assumed to be uniformly benign; in a retrospective propensity score-matched study, probiotic supplementation was not associated with improved eGFR and was associated with a higher occurrence of CMV viremia [109,111]. Although this observational association does not prove that probiotics caused CMV viremia, it reinforces the need for controlled transplant-specific safety data before routine supplementation can be recommended.

For these reasons, individualized nutrition remains more clinically applicable than universal microbiota supplementation. Dietary counseling in KTR should consider eGFR, potassium and phosphorus balance, protein requirements, body composition, PTDM risk, gastrointestinal tolerance, antibiotic exposure, history of *C. difficile* infection, infection risk, immunosuppressive regimen, and cardiovascular comorbidity. In stable recipients, increasing dietary fiber, improving protein quality, reducing excessive red and processed meat intake, and adopting Mediterranean-style or plant-forward dietary patterns may be reasonable strategies to support microbial metabolic profiles while also addressing established cardiometabolic goals. In recipients with active infection, severe diarrhea, recent rejection treatment, profound immunosuppression, poor graft function, or malnutrition, dietary or microbiota-targeted interventions should be more cautious and closely monitored [103,106]. Thus, nutrition-based modulation of the gut microbiota should be integrated with standard transplant dietary care rather than treated as an isolated microbiome intervention.

Overall, probiotics, prebiotics, and synbiotics should currently be viewed as investigational adjuncts rather than routine components of post-transplant care. Their future role will depend on well-designed transplant-specific trials that integrate dietary assessment, microbiome profiling, metabolomics, infection surveillance, immunosuppressive drug exposure, safety endpoints, and patient- and graft-centered outcomes. Until such evidence is available, nutritional modulation of the gut microbiota in KTR should prioritize individualized dietary patterns with established cardiometabolic benefits, while microbiota supplements should be considered only with attention to indication, safety, and clinical context [99,109]. This approach aligns microbiota-related hypotheses with evidence-based nutritional management and avoids premature translation of associative or mechanistic findings into routine clinical practice.

## 9. Diagnostic and Therapeutic Perspectives

Microbiome-based diagnostic and therapeutic strategies are not currently part of standard care in kidney transplantation. Gut microbiota profiling, microbial metabolite assessment, and pharmacomicrobiomic approaches are being investigated as potential tools for risk stratification, individualized immunosuppressive management, and biomarker-guided monitoring. However, microbiota-modifying interventions, including probiotics, prebiotics, synbiotics, and FMT, remain investigational in KTR and require cautious evaluation because this population is chronically immunosuppressed and frequently exposed to antibiotics, infections, metabolic complications, and variable graft function [1,35]. Therefore, the diagnostic and therapeutic approaches discussed below should be interpreted as translational research directions rather than clinically validated strategies. Their potential implementation requires standardized sampling, external validation, transplant-specific safety assessment, and demonstration of added value beyond established clinical, immunological, pharmacological, and graft-function markers.

### 9.1. Microbiome-Based Diagnostics and Biomarker-Guided Monitoring

Gut microbiome and metabolite signatures are being explored as candidate biomarkers of graft instability, rejection risk, and post-transplant complications. This approach is biologically plausible because microbial composition and metabolic output may reflect intestinal barrier function, inflammatory tone, immune regulation, antimicrobial exposure, diet, and residual graft function. Nevertheless, microbiome-based biomarkers remain exploratory because available studies differ in sampling time points, sequencing platforms, metabolomic methods, outcome definitions, and adjustment for confounders [1,9]. In addition, candidate signatures may be influenced by antibiotics, immunosuppressive regimen, diarrhea, infection, hospitalization, diet, diabetes, renal function, proteinuria, and time after transplantation. These variables should be systematically collected and adjusted for before microbiome-based markers can be considered clinically interpretable.

Cho et al. investigated gut microbiome and metabolite signatures in relation to acute kidney transplant rejection using stool and blood samples collected before transplantation and at 3 and 12 months after transplantation. Gut microbiota and metabolite profiles were assessed using 16S ribosomal ribonucleic acid (16S rRNA) sequencing and proton nuclear magnetic resonance (NMR) spectroscopy, and differences were observed between recipients who developed acute rejection and those without rejection [9]. These findings support the potential value of integrated microbiome–metabolome profiling, but candidate signatures require validation in larger independent cohorts and comparison with established clinical and immunological predictors, including serum creatinine, proteinuria, donor-specific antibodies, therapeutic drug monitoring, and biopsy-based assessment [1,9]. At present, such signatures should be regarded as candidate adjunctive biomarkers rather than replacements for standard rejection surveillance or biopsy-confirmed diagnosis.

### 9.2. Pharmacomicrobiomics and Tacrolimus Exposure

Pharmacomicrobiomics is particularly relevant to tacrolimus, a central immunosuppressive agent with a narrow therapeutic index and substantial pharmacokinetic variability. Inadequate exposure may increase rejection risk, whereas excessive exposure may contribute to nephrotoxicity, infection, metabolic complications, and other adverse effects; therefore, therapeutic drug monitoring remains essential [112].

Lee et al. reported an association between gut microbiota composition and tacrolimus dosing requirements in KTR, suggesting that intestinal microbial profiles may contribute to variability in tacrolimus exposure [10]. Additional mechanistic work supports microbiome–tacrolimus interactions, including evidence that gut microbiota may modulate tacrolimus pharmacokinetics through transcriptional regulation of intestinal ATP-binding cassette sub-family B member 1 (ABCB1) [96]. However, microbiome-associated tacrolimus variability should not be overinterpreted, because tacrolimus exposure is also influenced by CYP3A5 genotype, age, liver function, hematocrit, albumin, diarrhea, drug–drug interactions, adherence, time after transplantation, and graft function [112,113]. Nguyen et al. further showed that tacrolimus trough concentrations and time in the therapeutic range were associated with acute rejection risk early after renal transplantation, reinforcing that tacrolimus exposure is a dynamic clinical variable rather than a microbiome-specific effect [114]. Thus, microbiota-related variability should be evaluated within a broader pharmacokinetic framework that includes pharmacogenetics, clinical status, drug interactions, gastrointestinal symptoms, and therapeutic drug monitoring.

Therefore, pharmacomicrobiomic profiling may in the future complement therapeutic drug monitoring and pharmacogenetic approaches, but it cannot currently replace established tacrolimus monitoring. Prospective studies are needed to determine whether microbiome-informed models improve prediction of tacrolimus exposure, reduce time outside the therapeutic range, or improve clinically meaningful outcomes such as rejection, infection, nephrotoxicity, and long-term graft function [10,113]. Until such models are externally validated, microbiome-informed tacrolimus dosing should remain investigational.

### 9.3. Fecal Microbiota Transplantation

FMT is one of the most direct microbiota-modifying interventions, but its role in kidney transplantation remains uncertain. In CKD, Arteaga-Muller et al. reported that FMT was associated with changes in gut microbiota composition and stabilization of selected renal function parameters compared with placebo [115]. These findings suggest that microbiota manipulation may influence kidney-related metabolic or inflammatory pathways, but they should not be directly extrapolated to KTR because of differences in immunosuppression, infection risk, antimicrobial exposure, graft function, and clinical vulnerability [1,35]. Accordingly, FMT evidence from CKD should be treated as indirect proof-of-concept evidence rather than transplant-specific therapeutic evidence.

FMT should not be framed as a general strategy for restoring a presumed “normal” microbiota state or improving graft outcomes outside dedicated clinical studies. Broad microbial groups such as Bacteroidetes or Proteobacteria should not be uniformly classified as beneficial or anti-inflammatory because their clinical relevance depends on taxonomic resolution, strain-level features, functional capacity, and host context [1,115]. The most plausible near-term use of FMT in transplant populations is in selected infectious indications, particularly recurrent or severe CDI. Evidence from solid organ transplant recipients and real-world data supports FMT as a therapeutic approach for *C. difficile* infection, but this does not establish safety or efficacy for routine peri-transplant microbiome modulation [116,117]. Thus, FMT should be discussed as indication-specific therapy for selected recurrent or refractory CDI cases, not as a broad microbiome-restorative intervention in kidney transplantation.

Safety remains a major concern. The U.S. FDA has approved an orally administered fecal microbiota product for prevention of recurrent *C. difficile* infection in adults after antibacterial treatment, but this indication does not extend to broad microbiome restoration or graft-related outcomes [118]. FDA safety communications have also highlighted risks of serious infections due to pathogen transmission, including multidrug-resistant organisms, after investigational FMT [119,120]. Accordingly, FMT should currently be considered an indication-specific or experimental intervention in kidney transplantation, requiring trials that define patient selection, donor screening, timing, route of administration, microbiological safety monitoring, effects on immunosuppressive drug exposure, and clinically meaningful endpoints [116,120]. These safety considerations are particularly important in KTR because pathogen transmission, antimicrobial resistance, opportunistic infection, CMV reactivation, and drug exposure variability may have direct consequences for patient and graft outcomes.

### 9.4. Probiotics, Prebiotics, and Synbiotics

Probiotics, prebiotics, and synbiotics are attractive microbiota-targeted interventions because they are less invasive than FMT and may theoretically support commensal taxa involved in intestinal homeostasis, increase SCFA production, reduce uremic toxin generation, and improve intestinal barrier function. In transplant recipients, however, their effects are likely to be strain-specific, dose-dependent, and influenced by antibiotic exposure, diet, immunosuppression, baseline microbiota composition, and graft function [1,111]. Therefore, they should not be presented as uniformly beneficial or broadly safe in immunosuppressed recipients.

Evidence from other solid organ transplant populations suggests that probiotic or synbiotic interventions may reduce infectious complications in selected settings, including liver transplantation, but these findings should not be directly generalized to KTR because of differences in underlying disease, surgical context, immunosuppressive regimens, infection patterns, and microbiome dynamics [1,35]. In KTR, *Lactobacillus*-containing preparations have been discussed for antibiotic-associated diarrhea or *C. difficile*-related risk, but current evidence is insufficient to conclude that they reliably prevent or treat diarrhea, prevent *C. difficile* infection, or improve graft outcomes [1,111]. Thus, evidence from other solid organ transplant settings should be considered supportive but indirect.

Synbiotic supplementation has also been explored in KTR. Guida et al. reported that a short-course synbiotic intervention reduced plasma *p*-cresol concentrations, suggesting that modulation of gut microbial metabolism may influence uremic toxin-related pathways after transplantation [110]. Because *p*-cresol and related compounds such as *p*-cresyl sulfate are linked to host–microbe metabolism, intestinal dysbiosis, and systemic inflammation in kidney disease, this finding is mechanistically relevant but does not establish improvement in graft function or long-term transplant outcomes [44,110]. Moreover, *p*-cresol concentrations are influenced by diet, microbial metabolism, hepatic processing, renal clearance, and graft function, so changes in this biomarker should not be equated with clinical benefit. In contrast, Jang et al. found no clear improvement in kidney function among recipients receiving probiotic supplementation, while an increased risk of CMV infection was observed in the probiotic group [111]. Overall, probiotics, prebiotics, and synbiotics should currently be considered promising but unproven strategies, and future trials should define formulation, dose, timing, safety monitoring, and clinically meaningful endpoints [1,111]. The association between probiotic supplementation and CMV infection should be interpreted cautiously because of the observational design, but it underscores the need for transplant-specific safety monitoring.

### 9.5. Current Barriers to Clinical Translation

Several barriers limit the clinical implementation of microbiome-based diagnostics and therapies in kidney transplantation. Microbiome composition is highly variable and influenced by diet, geography, antibiotic exposure, hospitalization, immunosuppressive regimens, gastrointestinal symptoms, metabolic status, and graft function. Analytical approaches also remain heterogeneous, including differences in sample type, storage conditions, sequencing methods, metabolomic platforms, bioinformatic pipelines, and taxonomic resolution [1,9]. In addition, many studies are limited by small sample size, single-center design, variable timing of sampling, short follow-up, and incomplete adjustment for key clinical confounders. These limitations reduce reproducibility and make it difficult to define clinically actionable thresholds.

From a therapeutic perspective, safety is central. KTRs are chronically immunosuppressed, and interventions that introduce live microorganisms or profoundly alter microbial ecosystems may carry risks that are not fully captured by non-transplant studies. Therefore, microbiota-modifying interventions require transplant-specific safety evaluation, including monitoring for opportunistic infections, pathogen transmission, CMV infection or reactivation signals, antimicrobial resistance, drug exposure variability, and graft-related endpoints [111,120]. Future trials should also include standardized reporting of antibiotic exposure, immunosuppressive regimen, rejection treatment, infection events, diarrhea, diet, graft function, and adverse events.

Consequently, microbiome-based diagnostics and microbiota-targeted interventions should presently be viewed as promising translational research directions rather than established components of routine kidney transplant care. Their future role will depend on standardized methodology, prospective validation, and evidence that microbiome-informed approaches improve patient- or graft-centered outcomes [1,113]. To provide an integrated overview of the clinical evidence base discussed across the manuscript, Table 4 summarizes the main KTR studies evaluating gut microbiota, microbiota-associated metabolites, and microbiota-targeted interventions.

## 10. Future Directions and Conclusions

Current evidence links gut microbiota alterations and microbiota-derived metabolites with several biologically and clinically relevant aspects of kidney transplantation, including immune regulation, post-transplant complications, cardiovascular risk, graft function, and variability in immunosuppressive drug handling. In KTR, dysbiosis should be viewed as a dynamic phenotype shaped by both pre-transplant CKD-associated disturbances and post-transplant exposures, including hospitalization, antibiotics, immunosuppression, infection, diet, and graft function. Mechanistically, microbiota-related pathways may influence transplant biology through intestinal barrier dysfunction, microbial translocation, innate immune activation, altered Treg/Th17 balance, microbial metabolite signaling, uremic toxin generation, and endothelial stress. However, most mechanistic pathways remain incompletely validated in KTR cohorts, and many conclusions are extrapolated from CKD, dialysis, non-transplant inflammatory disease, solid organ transplant, or experimental studies. Therefore, microbiota-related alterations should be interpreted as biologically plausible modifiers or correlates rather than established causal determinants of graft outcomes.

Future research should move beyond descriptive microbiome profiling toward longitudinal multi-omics approaches integrating microbial taxonomy, metagenomic functional potential, metabolomics, immunophenotyping, drug exposure data, and biopsy-based graft outcomes. Serial sampling before transplantation and throughout the post-transplant course will be essential to distinguish transient perioperative dysbiosis from persistent microbial patterns associated with clinically meaningful transplant endpoints [4,121,122]. Integration of microbiome data with metabolomic readouts is particularly important because microbial metabolites may provide functional signals reflecting intestinal barrier integrity, immune regulation, uremic toxin generation, and host–microbe metabolic interactions that cannot be captured by taxonomic profiling alone [123,124]. Such studies should also systematically capture diet, antibiotic exposure, immunosuppressive regimen, diarrhea, infection, hospitalization, diabetes, renal function, proteinuria, graft function, and time after transplantation, because these factors may strongly confound microbiome–outcome associations.

An important translational objective is to determine whether microbiome-derived signals can improve risk stratification beyond established clinical, immunological, and histological monitoring tools. Future studies should prioritize reproducibility across independent cohorts, harmonization of sampling and sequencing strategies, and prospective evaluation of whether microbiome-derived markers improve prediction of rejection, graft dysfunction, infection risk, metabolic complications, or variability in immunosuppressive drug exposure [9,122]. Microbiota-derived metabolites should also be evaluated within a broader biomarker framework that includes renal clearance, nutritional status, mineral metabolism, endothelial injury, inflammation, proteinuria, and graft function. Data-driven analytical approaches and pharmacomicrobiomic models may support integration of clinical, laboratory, imaging, drug exposure, immunological, and omics-derived information, but their practical role in kidney transplant monitoring remains to be established [96,113,125,126,127]. At present, these tools should be considered hypothesis-generating and investigational rather than ready for routine clinical decision-making.

Microbiota-targeted interventions, including dietary modification, prebiotics, probiotics, synbiotics, postbiotics, and FMT, remain promising but insufficiently validated in kidney transplantation. These approaches are biologically plausible because they may influence microbial metabolite production, intestinal barrier integrity, immune regulation, and uremic toxin generation; however, findings from CKD or non-transplant populations cannot be directly extrapolated to immunosuppressed transplant recipients without dedicated safety and efficacy studies [8,128]. Probiotic and synbiotic strategies require particular caution, as available transplant-specific evidence does not establish graft benefit and indicates the need for careful safety monitoring [8,111,124]. Dietary strategies may be more scalable, but post-transplant nutritional management must account for graft function, metabolic complications, cardiovascular risk, infection susceptibility, electrolyte balance, and immunosuppressive therapy [8,129,130,131,132,133]. Therefore, nutritional modulation of the gut microbiota should be integrated with individualized transplant dietary care rather than treated as a stand-alone microbiome therapy.

Overall, microbiome-based diagnostics, pharmacomicrobiomic approaches, and microbiota-targeted interventions should currently be regarded as promising research directions rather than established components of routine kidney transplant care. Their translation will require standardized sampling and analytical methods, longitudinal multicenter studies, integration with metabolomics and immunophenotyping, careful adjustment for clinical confounders, and prospective trials assessing patient- and graft-centered outcomes [4,8,113,134]. Until such evidence is available, gut microbiota signatures and microbial metabolites should be considered potential modifiers and biomarkers of transplant-related risk rather than validated therapeutic targets. In conclusion, direct KTR studies support associations between gut microbiota features, microbiota-associated metabolites, and selected transplant outcomes, but the current evidence base remains predominantly observational and heterogeneous. Complementary evidence from CKD, dialysis, non-gut microbial compartments, other solid organ transplant populations, and experimental models provides biological plausibility but should not be interpreted as direct proof of causality in kidney transplantation. Future progress will depend on well-controlled, longitudinal, transplant-specific studies that determine whether microbiome-related markers add clinically meaningful value beyond established clinical, immunological, histological, nutritional, vascular, and graft-function biomarkers.

## 11. Strengths and Limitations

This review synthesizes clinical, experimental, and multi-omics evidence linking gut microbiota alterations and microbiota-derived metabolites with kidney transplantation, including immune tolerance, post-transplant complications, cardiovascular risk, graft function, and diagnostic or therapeutic perspectives. A key strength is the integrated transplant-focused framework, which considers both pre-transplant CKD-associated dysbiosis and post-transplant exposures such as hospitalization, antibiotics, immunosuppression, infection, diet, and graft function. This approach allows microbiota-related findings to be interpreted within the complex clinical context of KTR rather than as isolated microbial associations. Another strength is the cautious interpretation of the evidence, with emphasis on the distinction between association, biological plausibility, mechanistic inference, and clinical validation. The review also explicitly distinguishes direct KTR evidence from complementary non-gut microbiome evidence, indirect evidence from CKD, dialysis, or other solid organ transplant populations, and mechanistic or preclinical evidence. This distinction is important because many biologically plausible pathways remain insufficiently validated in transplant-specific clinical cohorts. In addition, the review incorporates methodological quality assessment and summarizes key study-level limitations to contextualize the strength of the available evidence.

Several limitations should be acknowledged. Most human studies remain observational, often cross-sectional, and limited by small sample sizes, which restricts causal inference and may reduce reproducibility. Substantial heterogeneity exists across cohorts, sampling time points, sequencing platforms, metabolomic methods, bioinformatic pipelines, taxonomic resolution, and clinical endpoint definitions. Important confounders, including diet, antibiotic exposure, immunosuppressive regimen, tacrolimus variability, infection, diarrhea, comorbidities, dialysis history, and residual graft function, are not uniformly captured or adjusted for across studies. Because of this heterogeneity, a quantitative meta-analysis was not feasible, and the evidence was synthesized narratively. Although methodological quality was assessed using design-specific critical appraisal tools, quality ratings were used to contextualize the evidence rather than to exclude studies, which is appropriate for the broad scope of this review but limits the strength of formal evidence grading. In particular, detailed dietary intake, including fiber, protein source, red and processed meat, choline, L-carnitine, and overall dietary pattern, was inconsistently assessed across studies, limiting interpretation of diet–microbiota–metabolite relationships. These factors may influence both microbiota composition and transplant outcomes, making it difficult to determine whether dysbiosis is a cause, consequence, or marker of post-transplant complications.

An additional limitation is the incomplete reporting of the underlying causes of CKD and ESKD in several transplant cohorts. This is relevant because autoimmune or immune-mediated kidney diseases, such as lupus nephritis or glomerulonephritis, may differ from non-immune causes of kidney failure in baseline inflammatory status, immune activation, immunosuppressive exposure before transplantation, and potentially gut microbiota composition. However, the etiology of kidney failure and background immunological disorders were not consistently reported or adjusted for across the available studies. Therefore, the influence of these factors could not be systematically evaluated in this review and should be considered an important limitation when interpreting microbiota-related associations in KTR. Similarly, information on induction therapy, maintenance immunosuppression, rejection treatment, antimicrobial prophylaxis, and recent antibiotic exposure was not consistently available across studies, although these factors may substantially influence microbiota composition, metabolite concentrations, infection risk, and graft outcomes.

Another limitation is that not all microbial compartments and metabolites directly reflect gut microbiota activity. Studies assessing salivary or urinary microbiota may provide clinically relevant information, but they should be interpreted as complementary evidence rather than direct evidence of gut dysbiosis. Similarly, circulating metabolites such as TMAO, indoxyl sulfate, and *p*-cresyl sulfate are influenced by microbial metabolism, diet, hepatic metabolism, renal clearance, and graft function. Therefore, these metabolites should be interpreted as host–microbe co-metabolites or biomarker signals rather than direct measures of microbial production alone. Experimental models provide mechanistic insights, but may not fully translate to immunosuppressed KTR. Finally, microbiota-targeted interventions remain insufficiently validated. Future work should prioritize longitudinal multicenter cohorts, standardized multi-omics workflows, harmonized clinical endpoints, careful adjustment for confounders, and interventional trials assessing patient- and graft-centered outcomes before microbiome-based tools can be incorporated into routine transplant care. Until such evidence is available, conclusions regarding microbiome-based diagnostics, pharmacomicrobiomics, dietary modulation, probiotics, synbiotics, and FMT should remain cautious and should not be interpreted as recommendations for routine clinical implementation.

## Figures and Tables

**Table 1:** Selected microbiota-related taxa and microbial groups and their potential relevance in kidney transplantation.

Taxon/Microbial Group	Direction or Pattern Described	Evidence Source	Potential Relevance or Interpretation	References
*Faecalibacterium*	Reduced abundance as part of depletion of SCFA-producing taxa	Direct KTR evidence and supportive non-transplant microbiota evidence	May indicate reduced abundance of taxa involved in SCFA production and intestinal metabolic homeostasis; clinical significance in KTR requires further validation.	[11,20,24]
*Roseburia*	Reduced abundance as part of depletion of SCFA-producing taxa	Direct KTR evidence and supportive non-transplant microbiota evidence	May reflect reduced capacity for fiber fermentation and SCFA production; effects are context-dependent and not uniformly protective.	[11,20,24]
Lachnospiraceae	Reduced abundance among SCFA-producing taxa	Direct KTR evidence and supportive non-transplant microbiota evidence	May indicate altered fiber fermentation and SCFA-related immunometabolic pathways, although transplant-specific outcome validation remains limited.	[11,20,24]
Ruminococcaceae	SCFA-producing family discussed as relevant to intestinal and metabolic homeostasis; transplant-specific direction is less consistently defined	Mainly supportive mechanistic/general microbiota evidence	May support SCFA production and intestinal homeostasis, although transplant-specific clinical validation remains limited.	[24]
Proteobacteria	Enrichment reported in KTR	Direct KTR evidence	May indicate microbiota instability and expansion of potentially opportunistic taxa; should not be interpreted as direct evidence of causality.	[11,24]
Urease-producing bacteria	Enrichment associated with CKD-related and post-transplant dysbiosis	Indirect CKD/dialysis evidence with possible relevance to KTR	May contribute to intestinal ammonia production, barrier dysfunction, and uremia-related metabolic disturbances, particularly when graft function remains impaired.	[13,14]
Proteolytic bacteria	Enrichment associated with CKD-related and post-transplant dysbiosis	Indirect CKD/dialysis evidence with possible relevance to KTR	May increase generation of uremic toxin precursors, including indoxyl sulfate and *p*-cresyl sulfate; circulating concentrations also depend on renal clearance and host metabolism.	[13,14]
Opportunistic or antibiotic-resistant taxa	Expansion may occur after antibiotic exposure, hospitalization, immunosuppression, and loss of colonization resistance	Direct and indirect transplant evidence	May reflect treatment-related ecological pressure and vulnerability to infectious complications; infection risk remains multifactorial.	[1,25,26,27]
*Clostridioides difficile*	Clinically relevant pathogen after disruption of the intestinal microbial ecosystem	Direct and indirect transplant evidence	May cause post-transplant diarrhea and contribute to dehydration, graft dysfunction, and morbidity; should be considered one cause among several gastrointestinal complications.	[26,28,29]
Gut-derived uropathogens	The gut may act as a reservoir for uropathogens	Direct and indirect transplant evidence	May contribute to UTI risk, although the pathway is multifactorial and should not be interpreted as direct causality.	[30,31,32]

Abbreviations: CKD, chronic kidney disease; KTR, kidney transplant recipients; SCFA, short-chain fatty acid; UTI, urinary tract infection.

**Table 2:** Selected post-transplant complications and microbiota-related findings in KTR.

Study	Related Complication	Microbiota-Related Finding/Interpretation
Xiang et al. [65]	Delayed graft function (DGF)	Early salivary microbiota profiles were associated with DGF risk, suggesting potential prognostic relevance of host-associated microbial signatures. This should not be interpreted as direct evidence that gut dysbiosis predicts DGF.
Lee et al. [25]	Post-transplant diarrhea	Gut microbiota dysbiosis was associated with diarrhea in KTR, supporting a link between microbial ecosystem disruption and gastrointestinal symptoms. Directionality remains uncertain because diarrhea, antibiotics, infection, diet, and immunosuppression can all affect gut microbiota composition.
Moghaddam et al. [30]	UTI/DGF	Gut microbiota alterations were described in a small prospective cohort with early UTI and DGF events. Findings are preliminary and do not establish independent microbial predictors of these complications.
Ding et al. [28]	CDI	CDI in KTR was associated with adverse clinical outcomes and should be considered an important infectious cause of post-transplant diarrhea. These outcomes may also reflect antibiotic exposure, hospitalization, immunosuppression, and illness severity.
Li et al. [29]	CDI and FMT	FMT has been discussed as a potential option for recurrent or refractory CDI after kidney transplantation, but not as a general microbiota-restorative strategy or a validated approach to improve graft outcomes.
Nambiar et al. [27]	Post-transplant infections	Infections remain a major source of morbidity and mortality after kidney transplantation, reflecting immunosuppression, comorbidity, antimicrobial exposure, and opportunistic pathogens.
Giessing et al. [31]	UTI after transplantation	UTIs are common after kidney transplantation and are driven by multiple clinical and urological risk factors; the gut may act as a reservoir for uropathogens, but causality remains complex.
Gołębiewska et al. [32]	Asymptomatic bacteriuria	Treatment of asymptomatic bacteriuria has not consistently improved graft function or prognosis, supporting a cautious approach and antibiotic stewardship.

Abbreviations: DGF, delayed graft function; UTI, urinary tract infection; CDI, *Clostridioides difficile* infection; FMT, fecal microbiota transplantation.

**Table 3:** Original studies and systematic evidence informing nutritional modulation of the gut microbiota after kidney transplantation.

Study	Study Design	Area/Topic	Evidence Source	Key Findings	Relevance for Kidney Transplant Recipients
Wu et al. 2020 [34]	Original experimental transplant model	SCFAs and kidney allograft tolerance	Mechanistic/preclinical transplant evidence	In experimental kidney transplantation, a high-fiber diet or acetate supplementation promoted donor-specific tolerance through Treg induction.	Supports the biological plausibility of a diet–microbiota–SCFA–immune regulation axis, although clinical validation in KTR is needed.
Wang et al. 2019 [100]	Original controlled dietary intervention study	Red meat and TMAO	Supportive non-transplant dietary intervention evidence	Chronic red meat intake increased circulating TMAO compared with white meat or non-meat protein sources.	Supports moderation of red meat intake when considering diet–microbiota–TMAO pathways, but the study was not conducted in KTR.
Flores-Guerrero et al. 2021 [92]	Original prospective cohort study	TMAO and graft failure	Direct KTR evidence	Higher circulating TMAO and its dietary determinants were associated with increased risk of kidney graft failure in renal transplant recipients.	Provides transplant-specific evidence linking diet-related TMAO pathways with adverse graft outcomes, but causality remains unproven.
Gomes-Neto et al. 2020 [104]	Original prospective cohort study	Mediterranean-style diet	Direct KTR nutritional evidence	Greater adherence to a Mediterranean-style diet was associated with lower risk of kidney function loss in KTR.	Provides direct transplant-specific evidence supporting Mediterranean-style diet as a practical long-term dietary framework; microbiota mediation was not established.
Osté et al. 2017 [105]	Original prospective cohort study	Mediterranean-style diet and PTDM	Direct KTR nutritional evidence	Mediterranean-style diet was associated with lower risk of new-onset diabetes after renal transplantation.	Relevant to prevention of PTDM and cardiometabolic complications after transplantation, but not direct evidence of microbiota-mediated benefit.
Cooper et al. 2022 [109]	Cochrane systematic review	Probiotics, prebiotics, and synbiotics in SOT	Broader SOT systematic evidence	Evidence was insufficient to support or refute routine use of synbiotics, prebiotics, or probiotics in SOT recipients.	Supports cautious, individualized use of microbiota supplements in immunosuppressed recipients.
Guida et al. 2017 [110]	Original pilot interventional study	Synbiotics and *p*-cresol	Direct KTR interventional evidence	Short-course synbiotic treatment reduced plasma *p*-cresol concentrations in KTR.	Provides proof-of-concept that microbiota-targeted nutritional interventions may influence selected uremic toxin pathways, but not evidence of improved graft outcomes.
Jang et al. 2025 [111]	Original retrospective propensity score-matched study	Probiotics and kidney transplant outcomes	Direct KTR observational safety/outcome evidence	Probiotic supplementation was not associated with improved eGFR and was associated with increased CMV viremia.	Highlights the need for safety monitoring and avoidance of routine unsupervised probiotic use in KTR.
Zeng et al. 2021 [102]	Systematic review and meta-analysis	TMAO and kidney function	Indirect CKD/general kidney-function evidence	Higher TMAO concentrations were associated with poorer kidney function.	Supports interpretation of TMAO as a kidney function-related metabolite, but evidence is not transplant-specific.

Abbreviations: CMV, cytomegalovirus; eGFR, estimated glomerular filtration rate; KTR, kidney transplant recipients; PTDM, post-transplant diabetes mellitus; SCFAs, short-chain fatty acids; SOT, solid organ transplantation; TMAO, trimethylamine *N*-oxide; Treg, regulatory T cell.

**Table 4:** Key clinical studies evaluating gut microbiota, microbiota-associated metabolites, and microbiota-targeted interventions in KTR.

Study	Study Design	Sample Size	Timing After Transplantation	Sample Type	Microbiome/ Metabolomic Method	Clinical Outcome	Main Finding	Adjustment for Confounders	Major Limitations
Lee et al. [10]	Pilot observational cohort	19 KTR	Serial sampling during the first month after transplantation	Stool	16S rRNA V4–V5 sequencing	Tacrolimus dosing requirements	Fecal *Faecalibacterium prausnitzii* abundance during the first post-transplant week was associated with tacrolimus dose requirement at 1 month.	Multivariable analysis included age and week-1 hemoglobin concentration.	Small pilot cohort; no CYP3A5 genotyping; dietary intake was not systematically collected; residual confounding by drug interactions, diarrhea, prophylactic medications, graft function, and time after transplantation remains possible.
Lee et al. [25]	Prospective observational cohort	71 KTR	Serial fecal sampling during the first 3 months after transplantation	Stool	16S rRNA gene sequencing	Post-transplant diarrhea	Post-transplant diarrhea was associated with reduced microbial diversity and gut microbiota dysbiosis.	Limited; clinical factors were considered, but residual confounding by diarrhea itself, antibiotics, infection, diet, and immunosuppression remains likely.	Directionality cannot be established; diarrhea may be both a cause and consequence of microbiota changes; early post-transplant sampling only.
Cho et al. [9]	Prospective observational study	97 KTR	Pre-transplantation, 3 months, and 12 months post-transplantation	Stool and blood	16S rRNA sequencing and fecal NMR-based metabolomics	Acute rejection	Thirty-three recipients developed acute rejection; recipients with acute rejection had reduced bacterial richness and diversity, and combined microbial, metabolomic, and clinical features improved prediction of acute rejection.	Clinical parameters were incorporated into prediction models.	Requires external validation; possible residual confounding by immunosuppression, antibiotics, diet, graft function, infection, and time after transplantation.
Holle et al. [7]	Multicenter prospective observational study	245 individuals, including 217 KT recipients; 562 fecal samples	Longitudinal post-transplant sampling	Stool	16S rRNA gene amplicon sequencing	Graft rejection	Gut microbiome alterations, including reduced diversity and lower abundance of SCFA-producing taxa, were detectable before clinically overt rejection.	Partial; longitudinal design supports temporal assessment, but residual clinical confounding remains possible.	Observational design; microbiome signatures require independent validation; causality and clinical utility remain unproven.
Wang et al. [85]	Case–control study	24 KTR with AMR and 29 stable KTR controls	Established post-transplant recipients	Stool	16S rRNA sequencing using Illumina MiSeq	AMR	AMR was associated with altered gut microbiota composition and reduced microbial richness compared with stable controls.	Limited; group comparisons were performed.	Cross-sectional design; small sample size; limited ability to determine whether dysbiosis precedes or follows rejection; treatment exposure, antibiotics, diet, infection, and graft function may confound results.
Wang et al. [87]	Case–control fecal metabolomics study	30 KTR with AMR, 35 KTR with stable renal function, and 21 ESRD controls	Established post-transplant recipients	Fecal samples	Untargeted LC-MS-based fecal metabolomics	AMR	AMR was associated with a distinct intestinal metabolic profile compared with ESRD and stable KTR controls.	Limited; comparisons included stable KTR and ESRD control groups.	Cross-sectional design; metabolite differences may reflect rejection, treatment exposure, renal clearance, diet, inflammation, or graft function.
Kim et al. [88]	Observational living donor kidney transplantation cohort	67 donor–recipient pairs	Early post-transplant follow-up, including assessment of early graft function	Stool	16S rRNA sequencing; donor–recipient microbiota similarity analysis using weighted UniFrac distance	Early allograft function	Greater donor–recipient gut microbiota similarity was associated with better early graft function.	Limited; clinical variables were considered, but shared environmental and dietary exposures may confound the association.	Living donor setting; limited generalizability to deceased donor transplantation; observational design; causality cannot be inferred.
Moghaddam et al. [30]	Preliminary prospective study	15 KTR	Early post-transplant period	Stool	Quantitative microbial assessment	UTI and DGF	Gut microbiota alterations were described in KTR; 3 recipients developed UTI and 2 recipients developed DGF.	Limited; low event numbers precluded robust adjustment.	Very small cohort; preliminary findings; no independent predictive model for UTI or DGF.
Xiang et al. [65]	Retrospective case–control study	40 KT recipients: 5 with DGF and 35 with immediate graft function	Perioperative period; salivary microbiota assessed early after transplantation, including day 1	Saliva	16S rRNA gene sequencing; random forest and logistic regression analyses	DGF	Salivary microbiota on day 1 after transplantation distinguished DGF from immediate graft function; model performance reported AUC 0.85 in random forest analysis.	Predictive modeling was performed.	Salivary microbiota, not direct gut microbiota evidence; small DGF subgroup; DGF is strongly influenced by donor, ischemia, perioperative, and graft-related factors.
Flores-Guerrero et al. [92]	Prospective cohort study	448 stable renal transplant recipients	Functioning graft at least 1 year after transplantation; median follow-up 5.3 years	Plasma and dietary data	Plasma TMAO measurement; dietary determinant assessment	Kidney graft failure	Higher plasma TMAO and its dietary determinants were associated with increased risk of graft failure; 58 recipients developed graft failure.	Extensive Cox regression adjustment including age, sex, BMI, blood pressure, lipids, albuminuria, eGFR, immunosuppressive medication, HLA mismatch, and dietary factors.	Observational biomarker study; TMAO reflects diet–microbiota–host metabolism and renal clearance; residual confounding remains possible.
Yepes-Calderón et al. [93]	Prospective study including two KTR cohorts	Cohort A: 623 KTR; Cohort B: 544 KTR; 315 potential kidney donors as healthy controls	Cohort A: pre-transplantation and 3, 6, 12, and 24 months post-transplantation; Cohort B: functioning graft ≥1 year, median 7.4 years post-transplantation	Plasma	Proton NMR measurement of TMAO	All-cause mortality	Plasma TMAO decreased sharply after transplantation but remained higher than in healthy controls. Higher post-transplant plasma TMAO was independently associated with increased all-cause mortality in both cohorts.	Time-dependent coefficient Cox regression analyses were performed.	Observational biomarker study; TMAO reflects diet–microbiota–host metabolism and renal clearance; residual confounding by graft function, diet, inflammation, cardiovascular burden, and comorbidity remains possible.
Korytowska et al. [94]	Observational biomarker study	92 KTR	Stable post-transplant recipients	Saliva and urine	LC-MS/MS measurement of salivary and serum uremic toxins	Graft deterioration	Salivary indoxyl sulfate, particularly when combined with proteinuria, was evaluated as a non-invasive biomarker for predicting graft deterioration.	Partial; biomarker associations were assessed.	Salivary biomarker study, not direct gut microbiota evidence; indoxyl sulfate is influenced by microbial metabolism, diet, host metabolism, renal clearance, proteinuria, and graft function.
Xiang et al. [95]	Single-center observational pharmacomicrobiomic study	102 KT recipients	Tacrolimus intra-patient variability assessed during the first post-transplant month; clinical outcomes assessed during the first year	Fecal samples and clinical pharmacokinetic data	16S rRNA sequencing and untargeted metabolomics	Tacrolimus intra-patient variability and 1-year clinical outcomes	High and low tacrolimus intra-patient variability groups differed in beta diversity, differential taxa, and metabolomic profiles; tacrolimus variability was associated with post-transplant hyperuricemia and new-onset diabetes.	Clinical correlation analyses were performed; selected outcomes were assessed for independent associations.	Single-center observational study; tacrolimus variability is multifactorial and may be influenced by adherence, CYP3A5 genotype, diarrhea, drug interactions, liver function, dose changes, graft function, and time after transplantation.
Guida et al. [110]	Single-center, parallel-group, double-blind, randomized pilot interventional study	36 KTR	Transplant vintage >12 months; stable graft function; no acute rejection or infection in previous 3 months; 30-day intervention	Plasma	Synbiotic intervention; plasma *p*-cresol measured by high-performance liquid chromatography at baseline, 15 days, and 30 days	Uremic toxin pathway	Synbiotic treatment reduced plasma *p*-cresol by 40% after 15 days and 33% after 30 days, while levels remained stable in the placebo group.	Randomized placebo-controlled design.	Small pilot study; short duration; surrogate biomarker endpoint; no evidence of improved graft function, cardiovascular outcomes, graft survival, or long-term prognosis.

Abbreviations: AMR, antibody-mediated rejection; CYP3A5, cytochrome P450 3A5; DGF, delayed graft function; eGFR, estimated glomerular filtration rate; ESRD, end-stage renal disease; KT, kidney transplantation; KTR, kidney transplant recipients; LC-MS, liquid chromatography–mass spectrometry; LC-MS/MS, liquid chromatography–tandem mass spectrometry; NMR, nuclear magnetic resonance; SCFA, short-chain fatty acid; TMAO, trimethylamine *N*-oxide; UTI, urinary tract infection.

## Data Availability

No new data were created or analyzed in this study. Data sharing is not applicable to this article.

## Supplementary Materials

The following supporting information can be downloaded at: https://www.mdpi.com/article/10.3390/nu18132056/s1, Method S1. Complete search strategies. Table S1. Reporting of underlying kidney disease etiology and immunological background in selected kidney transplant recipient studies included in this review. Table S2. Methodological quality assessment of original studies included in the review.

## Abbreviations

The following abbreviations are used in this manuscript:

16S rRNA	16S ribosomal ribonucleic acid
ABCB1	ATP-binding cassette sub-family B member 1
AhR	aryl hydrocarbon receptor
AMR	antibody-mediated rejection
AP-1	activator protein 1
CDI	*Clostridioides difficile* infection
CKD	chronic kidney disease
CMV	Cytomegalovirus
CYP3A5	cytochrome P450 3A5
DGF	delayed graft function
eGFR	estimated glomerular filtration rate
ESKD	end-stage kidney disease
FDA	Food and Drug Administration
FMT	fecal microbiota transplantation
FOXP3	forkhead box P3
FXR	farnesoid X receptor
GPR41	free fatty acid receptor 3
GPR43	free fatty acid receptor 2
GPR109A	hydroxycarboxylic acid receptor 2
HDACs	histone deacetylases
IFN-β	interferon beta
IL	Interleukin
IL-22	interleukin-22
KTR	kidney transplant recipients
LPS	Lipopolysaccharide
MDR1	multidrug resistance protein 1
MyD88	myeloid differentiation primary response 88
NF-κB	nuclear factor kappa-light-chain-enhancer of activated B cells
NMR	nuclear magnetic resonance
PAD	peripheral arterial disease
PRRs	pattern-recognition receptors
PTDM	post-transplant diabetes mellitus
SCFA	short-chain fatty acid
SOT	solid organ transplantation
TGR5	Takeda G protein-coupled receptor 5
Th1	T helper cell type 1
Th17	T helper 17 cells
TLRs	Toll-like receptors
TLR4/MD-2	Toll-like receptor 4/myeloid differentiation factor-2
TMAO	trimethylamine *N*-oxide
TMA	Trimethylamine
TNF-α	tumor necrosis factor α
Treg	regulatory T cells
TRIF	TIR domain-containing adaptor inducing IFN-β
UTI	urinary tract infection
ZO-1	zonula occludens-1
