# Supplementary material for: Kidney Transplantation and the Gut–Kidney Axis: Microbial, Metabolic, and Nutritional Implications for Graft and Patient Outcomes
Source: Nutrients. 2026 Jun 24;18(13):2056. doi: 10.3390/nu18132056 (PMC13362717; doi:10.3390/nu18132056)
Supplement: Supplementary file 1 [file nutrients-18-02056-s001.zip › Supplementary Method S1.pdf]

Supplementary Method S1. Complete search strategies.

A structured literature search was conducted in PubMed, Scopus, and Web of Science from database inception to May 2026. The search strategy was designed to identify studies addressing associations between kidney transplantation, gut microbiota, microbiota-derived metabolites, nutritional factors, and transplant-related outcomes. The search was supplemented by targeted searches for specific clinical outcomes and mechanistic pathways discussed in the review. Searches were limited to publications available in English. Conference papers and letters to the editor were excluded during full-text assessment.

PubMed

The following search strategy was used in PubMed and adapted for targeted searches:

(("kidney transplantation"[Title/Abstract] OR "renal transplantation"[Title/Abstract] OR "kidney transplant recipients"[Title/Abstract] OR "renal transplant recipients"[Title/Abstract] OR "kidney transplant"[Title/Abstract] OR "renal transplant"[Title/Abstract])
AND
("gut microbiota"[Title/Abstract] OR "gut microbiome"[Title/Abstract] OR dysbiosis[Title/Abstract] OR metagenomics[Title/Abstract] OR "16S rRNA"[Title/Abstract] OR "microbial metabolites"[Title/Abstract] OR "intestinal barrier"[Title/Abstract] OR "microbial translocation"[Title/Abstract] OR "short-chain fatty acids"[Title/Abstract] OR SCFA[Title/Abstract] OR butyrate[Title/Abstract] OR propionate[Title/Abstract] OR acetate[Title/Abstract] OR TMAO[Title/Abstract] OR "trimethylamine N-oxide"[Title/Abstract] OR choline[Title/Abstract] OR carnitine[Title/Abstract] OR tryptophan[Title/Abstract] OR indole[Title/Abstract] OR kynurenine[Title/Abstract] OR "bile acids"[Title/Abstract] OR "uremic toxins"[Title/Abstract] OR "indoxyl sulfate"[Title/Abstract] OR "p-cresyl sulfate"[Title/Abstract] OR probiotics[Title/Abstract] OR prebiotics[Title/Abstract] OR synbiotics[Title/Abstract] OR "fecal microbiota transplantation"[Title/Abstract] OR nutrition[Title/Abstract] OR diet[Title/Abstract] OR "dietary fiber"[Title/Abstract] OR "Mediterranean diet"[Title/Abstract] OR "plant-based diet"[Title/Abstract] OR pharmacomicrobiomics[Title/Abstract]))

Additional PubMed searches combined kidney transplantation terms with specific outcomes:

(("kidney transplantation"[Title/Abstract] OR "renal transplantation"[Title/Abstract] OR "kidney transplant recipients"[Title/Abstract] OR "renal transplant recipients"[Title/Abstract])
AND
(diarrhea[Title/Abstract] OR "Clostridioides difficile"[Title/Abstract] OR "Clostridium difficile"[Title/Abstract] OR "urinary tract infection"[Title/Abstract] OR "delayed graft function"[Title/Abstract] OR rejection[Title/Abstract] OR "chronic allograft dysfunction"[Title/Abstract] OR "graft failure"[Title/Abstract] OR mortality[Title/Abstract] OR "cardiovascular disease"[Title/Abstract] OR "endothelial dysfunction"[Title/Abstract] OR "arterial stiffness"[Title/Abstract] OR "peripheral arterial disease"[Title/Abstract])
AND
("gut microbiota"[Title/Abstract] OR "gut microbiome"[Title/Abstract] OR dysbiosis[Title/Abstract] OR "microbial metabolites"[Title/Abstract] OR TMAO[Title/Abstract] OR "trimethylamine N-oxide"[Title/Abstract] OR "indoxyl sulfate"[Title/Abstract] OR "p-cresyl sulfate"[Title/Abstract]))

Scopus

The following search strategy was used in Scopus:

TITLE-ABS-KEY(("kidney transplantation" OR "renal transplantation" OR "kidney transplant recipients" OR "renal transplant recipients" OR "kidney transplant" OR "renal transplant")
AND
("gut microbiota" OR "gut microbiome" OR dysbiosis OR metagenomics OR "16S rRNA" OR "microbial metabolites" OR "intestinal barrier" OR "microbial translocation" OR "short-chain fatty acids" OR SCFA OR butyrate OR propionate OR acetate OR TMAO OR "trimethylamine N-oxide" OR choline OR carnitine OR tryptophan OR indole OR kynurenine OR "bile acids" OR "uremic toxins" OR "indoxyl sulfate" OR "p-cresyl sulfate" OR probiotics OR prebiotics OR synbiotics OR "fecal microbiota transplantation" OR nutrition OR diet OR "dietary fiber" OR "Mediterranean diet" OR "plant-based diet" OR pharmacomicrobiomics))

Additional Scopus searches combined kidney transplantation terms with outcome-specific terms:

TITLE-ABS-KEY(("kidney transplantation" OR "renal transplantation" OR "kidney transplant recipients" OR "renal transplant recipients")
AND
(diarrhea OR "Clostridioides difficile" OR "Clostridium difficile" OR "urinary tract infection" OR "delayed graft function" OR rejection OR "chronic allograft dysfunction" OR "graft failure" OR mortality OR "cardiovascular disease" OR "endothelial dysfunction" OR "arterial stiffness" OR "peripheral arterial disease")
AND
("gut microbiota" OR "gut microbiome" OR dysbiosis OR "microbial metabolites" OR TMAO OR "trimethylamine N-oxide" OR "indoxyl sulfate" OR "p-cresyl sulfate"))

Web of Science

The following search strategy was used in Web of Science:

TS=(("kidney transplantation" OR "renal transplantation" OR "kidney transplant recipients" OR "renal transplant recipients" OR "kidney transplant" OR "renal transplant")
AND
("gut microbiota" OR "gut microbiome" OR dysbiosis OR metagenomics OR "16S rRNA" OR "microbial metabolites" OR "intestinal barrier" OR "microbial translocation" OR "short-chain fatty acids" OR SCFA OR butyrate OR propionate OR acetate OR TMAO OR "trimethylamine N-oxide" OR choline OR carnitine OR tryptophan OR indole OR kynurenine OR "bile acids" OR "uremic toxins" OR "indoxyl sulfate" OR "p-cresyl sulfate" OR probiotics OR prebiotics OR synbiotics OR "fecal microbiota transplantation" OR nutrition OR diet OR "dietary fiber" OR "Mediterranean diet" OR "plant-based diet" OR pharmacomicrobiomics))

Additional Web of Science searches combined kidney transplantation terms with outcome-specific terms:

TS=(("kidney transplantation" OR "renal transplantation" OR "kidney transplant recipients" OR "renal transplant recipients")
AND
(diarrhea OR "Clostridioides difficile" OR "Clostridium difficile" OR "urinary tract infection" OR "delayed graft function" OR rejection OR "chronic allograft dysfunction" OR "graft failure" OR mortality OR "cardiovascular disease" OR "endothelial dysfunction" OR "arterial stiffness" OR "peripheral arterial disease")
AND
("gut microbiota" OR "gut microbiome" OR dysbiosis OR "microbial metabolites" OR TMAO OR "trimethylamine N-oxide" OR "indoxyl sulfate" OR "p-cresyl sulfate"))

Study selection

A total of 612 records were identified through database searching. After removal of 98 duplicate records, 514 records were screened by title and abstract. Of these, 322 records were excluded because they were not relevant based on title or abstract (n = 306), were not available in English (n = 12), or full text was not available (n = 4). Sixty-two reports were excluded at the full-text stage because they were not relevant to the review topic (n = 54), were letters to the editor (n = 5), or were conference papers (n = 3). Finally, 130 studies were included in the narrative synthesis.
