# Supplementary material for: Kidney Transplantation and the Gut–Kidney Axis: Microbial, Metabolic, and Nutritional Implications for Graft and Patient Outcomes
Source: Nutrients. 2026 Jun 24;18(13):2056. doi: 10.3390/nu18132056 (PMC13362717; doi:10.3390/nu18132056)
Supplement: Supplementary file 1 [file nutrients-18-02056-s001.zip › Supplementary Table S1.pdf]

Supplementary Table S1. Reporting of underlying kidney disease etiology and immunological background in selected kidney transplant recipient studies included in this review.

Study	Clinical focus	CKD/ESKD etiology and immunological background	Major confounders relevant to interpretation	Main relevance to this review
Lee et al. [25]	Post-transplant diarrhea and gut microbiota dysbiosis	Not sufficiently reported or stratified.	Diarrhea, antimicrobial exposure, mycophenolate-related gastrointestinal toxicity, and other post-transplant therapies.	Dysbiosis was associated with diarrhea in kidney transplant recipients, but interpretation is limited by treatment-related and clinical confounders.
Moghaddam et al. [30]	Gut microbiota, UTI, and DGF	Not sufficiently reported or stratified.	Small cohort size, early post-transplant clinical instability, UTI, antimicrobial exposure, and DGF.	Gut microbiota alterations were described in a small prospective cohort with early UTI and DGF events; findings remain preliminary.
Xiang et al. [65]	Early salivary microbiota and DGF	Not sufficiently reported or stratified.	Peri-transplant exposures, early graft injury, immunosuppression, and non-gut microbial sampling.	Findings should be interpreted as complementary evidence from a non-gut microbial compartment rather than direct gut microbiota evidence.
Cho et al. [9]	Microbiome/metabolome signatures and acute rejection	Not sufficiently reported or stratified.	Rejection-related immune activation, immunosuppressive treatment, antibiotic exposure, and clinical heterogeneity.	Microbiome–metabolome differences were observed in relation to acute rejection, but confounding by treatment and immune status remains possible.
Holle et al. [7]	Gut microbiome changes before clinically overt graft rejection	Not sufficiently reported or stratified.	Immunosuppressive exposure, antimicrobial exposure,	Gut microbiome alterations may precede rejection, but causality and

Study	Clinical focus	CKD/ESKD etiology and immunological background	Major confounders relevant to interpretation	Main relevance to this review
			inflammation, graft function, and other post-transplant clinical changes.	independence from clinical confounders remain uncertain.
Wang et al. [85]	Gut microbiota alterations associated with AMR	Not sufficiently reported or stratified.	Alloimmune risk, immunosuppressive treatment, antibiotic exposure, and metabolomic heterogeneity.	AMR was associated with altered gut microbial composition, but validation and adjustment for confounders are needed.
Kim et al. [88]	Donor–recipient gut microbiota similarity and graft function	Not sufficiently reported or stratified.	Donor–recipient factors, peri-transplant exposures, immunosuppression, antibiotic exposure, and early graft function.	Donor–recipient microbiota similarity was associated with early graft function, but underlying kidney disease etiology was not sufficiently addressed in the microbiota-related interpretation.
Lee et al. [10]	Gut microbiota and tacrolimus dosing requirements	Not sufficiently reported or stratified.	Tacrolimus exposure, immunosuppressive regimen, antibiotic exposure, and interindividual pharmacokinetic variability.	Gut microbiota composition was associated with tacrolimus dosing requirements, but microbiome-informed dosing remains investigational.
Guida et al. [110]	Short-course synbiotic treatment in kidney transplant recipients	Not sufficiently reported or stratified.	Immunosuppression, dietary intake, baseline clinical heterogeneity, and microbiota-modifying intervention.	Synbiotic treatment reduced plasma p-cresol concentrations, supporting proof-of-concept microbiota modulation.

Study	Clinical focus	CKD/ESKD etiology and immunological background	Major confounders relevant to interpretation	Main relevance to this review
Jang et al. [111]	Probiotic supplementation and kidney transplant outcomes	Not sufficiently reported or stratified.	Immunosuppression, infection risk, CMV infection, antibiotic exposure, and safety considerations.	Probiotic supplementation was not associated with improved kidney function and was associated with increased CMV infection risk.

"*Not sufficiently reported or stratified*" indicates that the available study information did not allow subgroup interpretation according to CKD/ESKD etiology or autoimmune/immune-mediated background. Abbreviations: AMR, antibody-mediated rejection; CKD, chronic kidney disease; CMV, cytomegalovirus; DGF, delayed graft function; ESKD, end-stage kidney disease; UTI, urinary tract infection.
