# Supplementary material for: Kidney Transplantation and the Gut–Kidney Axis: Microbial, Metabolic, and Nutritional Implications for Graft and Patient Outcomes
Source: Nutrients. 2026 Jun 24;18(13):2056. doi: 10.3390/nu18132056 (PMC13362717; doi:10.3390/nu18132056)
Supplement: Supplementary file 1 [file nutrients-18-02056-s001.zip › Supplementary Table S2.pdf]

Supplementary Table S2. Methodological quality assessment of original studies included in the review.

Study	Evidence type	Study design	JBI tool used	Main methodological concerns	Confounder adjustment	Overall methodological appraisal
Yang et al. [6]	Direct KTR metabolite evidence; TMAO and peripheral arterial disease	Observational cross-sectional association study	JBI Analytical Cross-Sectional Studies	Cross-sectional design limits temporal inference. TMAO concentrations may reflect diet, host metabolism, renal clearance, graft function, and comorbidity rather than microbial production alone.	Adjustment for selected cardiovascular and biochemical variables was reported, but residual confounding remains possible.	Moderate
Holle et al. [7]	Direct KTR gut microbiome evidence; rejection	Prospective/longitudinal observational cohort	JBI Cohort Studies	Observational design limits causal inference. Microbiome alterations preceding rejection may reflect inflammation, graft dysfunction, medication changes, antibiotic exposure, or dietary changes.	Adjustment for selected clinical variables was performed, but residual confounding remains possible.	Moderate to good
Cho et al. [9]	Direct KTR gut microbiome and metabolite evidence; acute rejection prediction	Prospective observational study	JBI Cohort Studies	Biomarker prediction requires external validation. Risk of overfitting and center-specific effects should be considered. Associations may be influenced by immunosuppression, antibiotics, diet, and graft function.	Multivariable modeling/adjustment for selected variables was reported, but residual confounding remains possible.	Moderate to good
Lee et al. [10]	Direct KTR gut microbiota evidence; tacrolimus dosing	Pilot observational study / early post-transplant cohort	JBI Cohort Studies	Small pilot study. Tacrolimus dosing is influenced by multiple non-microbial factors, including CYP3A5 genotype, liver function, drug interactions, adherence, diarrhea, and time after transplantation.	Limited adjustment for the full range of pharmacokinetic confounders.	Moderate
Voroneanu et al. [17]	Comparative evidence; hemodialysis and kidney transplant populations	Comparative cross-sectional observational study	JBI Analytical Cross-Sectional Studies	Comparative design may be affected by differences in renal function, dialysis exposure, diet, comorbidity, medication use, and timing after transplantation.	Adjustment for selected clinical factors was limited or incompletely reported.	Moderate
Fricke et al. [19]	Direct transplant microbiota evidence; peri-transplant microbiota characterization	Longitudinal observational study	JBI Cohort Studies	Small cohort and heterogeneous peri-transplant exposures. Antibiotics, hospitalization, surgery, immunosuppression, and sampling time may strongly affect microbiota composition.	Limited adjustment for major clinical confounders.	Moderate
Swarte et al. [21]	Mixed solid organ transplant evidence including KTR; gut microbiome and mortality	Observational cohort study	JBI Cohort Studies	Mixed transplant population introduces heterogeneity by organ type, immunosuppressive regimen, infection risk, and comorbidity. Mortality associations may reflect illness severity and non-microbial factors.	Multivariable adjustment was performed, although residual confounding remains possible.	Good
Choo et al. [22]	Direct KTR functional microbiota evidence; prebiotic starch fermentation capacity	Functional observational microbiota study	JBI Analytical Cross-Sectional Studies	Small study with surrogate functional endpoints. Fermentation capacity may be influenced by baseline diet, microbiota composition, medications, and graft function.	Limited adjustment for dietary and clinical confounders.	Low to moderate

Study	Evidence type	Study design	JBI tool used	Main methodological concerns	Confounder adjustment	Overall methodological appraisal
Swarte et al. [20]	Direct KTR gut microbiome evidence; dysbiosis	Cross-sectional observational comparison	JBI Analytical Cross-Sectional Studies	Cross-sectional design precludes temporal or causal inference. Results may be influenced by diet, medication, renal function, antibiotics, diabetes, and comorbidity.	Adjustment for selected clinical variables was reported, but residual confounding remains possible.	Moderate
Lee et al. [25]	Direct KTR gut microbiota evidence; diarrhea	Prospective observational study with serial fecal sampling and diarrhea comparison	JBI Cohort Studies	The relationship between diarrhea and dysbiosis may be bidirectional. Diarrhea, infections, antibiotics, diet, and mycophenolate exposure may all alter microbiota composition.	Adjustment for selected clinical variables was reported, but residual confounding remains likely.	Moderate
Moghaddam et al. [30]	Direct KTR gut microbiota evidence; UTI and DGF	Preliminary prospective study	JBI Cohort Studies	Small sample size and low number of clinical events limit statistical power. Early post-transplant microbiota is strongly affected by antibiotics, hospitalization, surgery, and graft function.	Limited adjustment because of small cohort size and few events.	Low to moderate
Swarte et al. [49]	Direct KTR gut microbiome evidence; health-related quality of life	Cross-sectional association study	JBI Analytical Cross-Sectional Studies	Patient-reported outcomes are multifactorial. Associations may be confounded by comorbidity, mental health, medications, diet, renal function, and socioeconomic factors.	Multivariable adjustment for selected variables was reported.	Moderate to good
Xiang et al. [65]	Complementary KTR salivary microbiota evidence; DGF prediction	Observational predictive cohort study	JBI Cohort Studies	Salivary rather than gut microbiota was assessed. Findings may reflect oral microbiota, perioperative status, antibiotic exposure, hospitalization, and systemic illness rather than gut dysbiosis.	Adjustment for selected predictors was reported, but external validation is required.	Moderate
Wang et al. [85]	Direct KTR gut microbiota evidence; antibody-mediated rejection	Case-control comparison	JBI Case-Control Studies	Case-control design limits temporal inference. Microbiota differences may reflect rejection, treatment, renal dysfunction, inflammation, antibiotics, or hospitalization.	Limited or partial adjustment for confounders.	Moderate
Visconti et al. [86]	Direct KTR gut microbiota evidence; graft rejection	Cross-sectional case-control study	JBI Case-Control Studies	Small sample size and cross-sectional comparison limit causal inference. Rejection phenotypes and treatment exposure may be heterogeneous.	Strict inclusion and exclusion criteria were used to address selected microbiota confounders; residual confounding remains possible.	Low to moderate
Wang et al. [87]	Direct KTR fecal metabolomic evidence; antibody-mediated rejection	Case-control / cross-sectional comparison	JBI Case-Control Studies	Fecal metabolic profiles may be influenced by diet, renal function, medications, inflammation, and treatment for rejection. Limited integration with longitudinal graft outcomes.	Limited or partial adjustment for confounders.	Moderate

Study	Evidence type	Study design	JBI tool used	Main methodological concerns	Confounder adjustment	Overall methodological appraisal
Kim et al. [88]	Direct KTR gut microbiota evidence; donor-recipient similarity and graft function	Observational cohort study in living donor kidney transplantation	JBI Cohort Studies	Living donor setting may limit generalizability to deceased donor transplantation. Donor-recipient relatedness, shared environment, diet, antibiotics, and immunosuppression may confound associations.	Adjustment for selected clinical variables was reported.	Moderate
Wu et al. [89]	Complementary urinary microbiome evidence; chronic allograft dysfunction	Case-control study	JBI Case-Control Studies	Urinary rather than gut microbiome was assessed. Urinary microbiota may be influenced by urological factors, sampling method, antimicrobial exposure, colonization, and infection.	Partial adjustment; residual confounding likely.	Moderate
Modena et al. [90]	Complementary urinary microbiome evidence; interstitial fibrosis/tubular atrophy	Longitudinal biopsy-linked observational study	JBI Cohort Studies	Urinary rather than gut microbiome was assessed. Small cohort and biopsy-linked associations do not establish causality. Sampling and urological confounders may affect results.	Partial adjustment; residual confounding possible.	Moderate
Flores-Guerrero et al. [92]	Direct KTR microbiota-associated metabolite evidence; TMAO and graft failure	Prospective cohort study	JBI Cohort Studies	TMAO reflects diet, gut microbial metabolism, hepatic metabolism, renal clearance, and graft function. Observational design limits causal interpretation.	Multivariable adjustment for several clinical and biochemical variables was reported.	Good
Yepes-Calderón et al. [93]	Direct KTR microbiota-associated metabolite evidence; TMAO and mortality	Prospective cohort study	JBI Cohort Studies	TMAO interpretation is limited by diet, renal clearance, host metabolism, graft function, and residual cardiovascular risk factors.	Multivariable adjustment was performed, but residual confounding remains possible.	Good
Korytowska et al. [94]	Complementary biomarker evidence; salivary indoxyl sulfate and graft deterioration	Observational biomarker study	JBI Analytical Cross-Sectional Studies	Salivary biomarker rather than gut microbiota was assessed. Indoxyl sulfate levels are affected by renal clearance, proteinuria, diet, host metabolism, and graft function.	Limited or partial adjustment for confounders.	Moderate
Xiang et al. [95]	Direct KTR gut microbiota/metabolite evidence; tacrolimus variability and one-year outcomes	Observational pharmacomicrobiomic cohort study	JBI Cohort Studies	Tacrolimus variability is influenced by dose, adherence, CYP3A5 genotype, liver function, drug interactions, diarrhea, diet, and time after transplantation.	Adjustment/modeling for selected variables was reported, but residual confounding remains likely.	Moderate
Degraeve et al. [96]	Translational pharmacomicrobiomic evidence; tacrolimus pharmacokinetics	Translational mechanistic study with human and preclinical components	Human component: JBI Analytical Cross-Sectional Studies; preclinical component not assessed using JBI human tools	Translational design limits direct clinical inference. Human component is likely small, while animal/in vitro data may not fully translate to KTR outcomes.	Limited clinical confounder adjustment.	Moderate for human component

Study	Evidence type	Study design	JBI tool used	Main methodological concerns	Confounder adjustment	Overall methodological appraisal
Gomes-Neto et al. [104]	Nutritional KTR evidence; Mediterranean diet and kidney function loss	Observational cohort study	JBI Cohort Studies	Dietary assessment may be affected by measurement error and changes over time. Associations may be confounded by lifestyle, socioeconomic factors, renal function, medication, and comorbidity.	Multivariable adjustment was reported.	Good
Osté et al. [105]	Nutritional KTR evidence; Mediterranean diet and new-onset diabetes after transplantation	Observational cohort study	JBI Cohort Studies	Dietary exposure and diabetes risk are influenced by weight, immunosuppression, baseline metabolic risk, physical activity, and lifestyle factors.	Multivariable adjustment was reported.	Good
Guida et al. [110]	Direct KTR intervention evidence; synbiotics and p-cresol	Pilot placebo-controlled randomized interventional study	JBI Randomized Controlled Trials	Short intervention duration and surrogate biomarker endpoint. Limited ability to infer long-term clinical effects on graft outcomes. Diet and renal clearance may affect p-cresol concentrations.	Randomized intervention design reduces some confounding, but adjustment for diet and clinical variables may be limited.	Moderate
Jang et al. [111]	Direct KTR probiotic exposure/intervention evidence; clinical outcomes	Retrospective propensity-score matched study	JBI Cohort Studies	Retrospective design and possible indication bias. Probiotic exposure may be associated with unmeasured clinical or behavioral factors despite matching.	Propensity-score matching was performed, but residual confounding remains possible.	Moderate
Nguyen et al. [114]	Clinical pharmacology evidence; tacrolimus exposure and acute rejection	Retrospective observational cohort study	JBI Cohort Studies	Does not directly assess microbiota. Relevant mainly as background evidence for tacrolimus variability and rejection risk. Confounding by adherence, dosing strategy, genotype, and clinical severity is possible.	Adjustment for selected clinical variables was reported.	Moderate
Cheng et al. [116]	Indirect/complementary SOT evidence; FMT for recurrent/severe CDI	Multicenter retrospective cohort / clinical experience	JBI Cohort Studies	Mixed solid organ transplant population. Evidence is indication-specific for CDI and should not be generalized to microbiota restoration or graft outcomes.	Multicenter design strengthens generalizability, but residual confounding and selection bias remain possible.	Moderate
Lan et al. [121]	Direct KTR gut microbiome/metabolome evidence; kidney function status	Cross-sectional observational comparison	JBI Analytical Cross-Sectional Studies	Cross-sectional design limits temporality. Metabolite concentrations may reflect graft function, renal clearance, diet, host metabolism, and medications.	Partial adjustment for clinical variables.	Moderate
Zhong et al. [122]	Direct KTR gut microbiota evidence; longitudinal dynamics	Prospective longitudinal cohort study	JBI Cohort Studies	Longitudinal design strengthens temporal assessment, but microbiota dynamics may still be influenced by antibiotics, diet, infection, immunosuppression, hospitalization, and time after transplantation.	Adjustment for selected variables was reported; residual confounding possible.	Moderate to good
Kouidhi et al. [123]	Direct KTR fecal metabolomic evidence; KTR vs healthy controls	Cross-sectional case-control comparison	JBI Case-Control Studies	Differences from healthy controls may reflect transplant status, renal function, diet, medications, comorbidity, and immunosuppression rather than microbiota-related pathways alone.	Limited or partial adjustment for confounders.	Moderate

Study	Evidence type	Study design	JBI tool used	Main methodological concerns	Confounder adjustment	Overall methodological appraisal
Dukaew et al. [127]	Direct KTR gut microbiota evidence; tacrolimus metabolism rate	Observational early post-transplant cohort study	JBI Cohort Studies	Tacrolimus metabolism is affected by genotype, dose, drug interactions, diarrhea, liver function, graft function, diet, and time after transplantation.	Adjustment for selected clinical factors was reported, but residual confounding remains likely.	Moderate
Sung et al. [130]	Direct KTR gut microbiome evidence; sickness symptoms	Prospective longitudinal observational study	JBI Cohort Studies	Symptom burden is multifactorial and may be influenced by medication adverse effects, infection, mental health, diet, graft function, and comorbidity.	Adjustment for selected variables was reported.	Moderate
Lecronier et al. [131]	Direct KTR gut microbiota evidence; post-transplant diabetes	Observational longitudinal study	JBI Cohort Studies	Post-transplant diabetes is multifactorial. Associations may be confounded by diet, weight gain, corticosteroids, calcineurin inhibitors, antibiotics, metabolic status, and graft function.	Partial adjustment for clinical variables.	Moderate

Overall methodological appraisal categories were assigned qualitatively based on the relevant Joanna Briggs Institute critical appraisal domains, including clarity of inclusion criteria, appropriateness of exposure and outcome assessment, identification and adjustment of confounders, completeness of follow-up where applicable, and adequacy of statistical analysis. These categories were used to contextualize the strength of evidence and were not intended as formal numerical JBI scores or exclusion criteria. Abbreviations: AMR, antibody-mediated rejection; CDI, Clostridioides difficile infection; DGF, delayed graft function; FMT, fecal microbiota transplantation; HRQoL, health-related quality of life; JBI, Joanna Briggs Institute; KTR, kidney transplant recipients; PAD, peripheral arterial disease; SOT, solid organ transplantation; TMAO, trimethylamine N-oxide; UTI, urinary tract infection.
